# Whisker‐Implanted Biomimetic Electronic Skin for Tactile Sensing and Blind Perception

**DOI:** 10.1002/advs.202408162

**Published:** 2024-11-05

**Authors:** Mohammad Zarei, An Woo Jeong, Seung Goo Lee

**Affiliations:** ^1^ Department of Chemistry University of Ulsan Ulsan 44610 South Korea

**Keywords:** electronic skins, human‐machine interfaces, pressure sensors, rodent whiskers, wearable electronics

## Abstract

Rodent whiskers are a distinct class of tactile sensors that work in conjunction with the biological skin to discern airstreams and obstacles with remarkable sensitivity, facilitating navigation around proximate objects. In this study, a flexible artificial skin is developed comprising sensory active units, including electronic skin (e‐skin) and an artificial whisker, inspired by the sensory capabilities of rodent skin and whiskers. As a novel strategy, unique congruent air pockets are introduced within the e‐skin to enhance the sensitivity. Mechanical stimuli applied to the artificial whisker are efficiently transmitted to the active e‐skin, which generates a sensitive tactile perception response. The developed artificial skin exhibits high sensitivity, a wide sensing range, high flexibility, superior stability, and tensile strength. The artificial whisker facilitates the sensitive detection of a broad range of applied mechanical forces. Therefore, the artificial skin can sense subtle and vigorous tactile stimuli including airstreams and field obstacles. The ability to sense, discriminate, and decipher the airstreams and obstacles imparts outstanding tactile sensing and blind perception characteristics to the artificial skin. This artificial skin is a promising platform for the development of sensitive e‐skins suitable for a broad range of applications, such as human‐machine interfaces, robotics, and wearable electronics.

## Graphical abstract

In this study, the development of a biomimetic artificial skin composed of active sensory units, including electronic skin and an artificial whisker, inspired by the sensory functions of rodents’ biological skin and whiskers is reported. The artificial whisker is capable of sensitive detection of both subtle and vigorous mechanical stimuli.

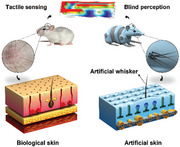

## 1 Introduction

Rodents can rapidly and precisely detect their surroundings using their highly sensitive whiskers.^[^ ^1^, ^2^ ^]^ Tactile information obtained from rodent whiskers is crucial for identifying obstacles, airstreams, and wind movements.^[^ ^3^, ^4^ ^]^ Rodent whiskers comprise numerous nerve endings that are incredibly sensitive to even the slightest air vibrations and mechanical stimuli and are deflected or displaced by coming in contact with surrounding air or passing over an airstream. Mechanoreceptors, which are specialized sensory cells located at the base of rodent whiskers, recognize movements and transmit information to the olfactory bulb, orbitofrontal cortex, and barrel cortex for processing. Based on this mechanism, rodents can identify airstream alterations and wind direction by perceiving the timing and deflection patterns of their whisker movements.^[^ ^5^, ^6^, ^7^ ^]^ Using this ability, rodents can locate objects, such as predators or barriers, facilitating their navigation and orientation.^[^ ^8^ ^]^ The ultrasensitive whiskers provide rodents with a detailed and precise perception even in low‐light scenarios where their visual capabilities are limited.

The development of smart and user‐interactive wearable electronics has led to a growing interest in creating functionalities that imitate biological sensing systems.^[^ ^9^, ^10^, ^11^, ^12^, ^13^, ^14^ ^]^ Recent advancements in surface engineering, novel sensing materials, and flexible active substrates have led to the development of artificial electronic skins (e‐skins).^[^ ^15^, ^16^, ^17^, ^18^, ^19^, ^20^, ^21^, ^22^, ^23^, ^24^, ^25^ ^]^ Artificial whiskers are another important class of sensory platforms that can be used for tactile sensing, airstream detection, motion balance, and spatial mapping of nearby objects for applications in advanced robotics, wearable sensors, and human‐machine interfaces (HMIs).^[^ ^26^, ^27^, ^28^, ^29^, ^30^, ^31^, ^32^, ^33^, ^34^ ^]^ However, previous studies on the fabrication of artificial whiskers involved bulky torque/force sensor designs that are unsuitable for integrated, lightweight, compact, sensitive, dynamic, and scalable platforms, which are essential for developing high‐performance e‐skins and wearable electronics for practical applications.

Several approaches have been employed for fabricating artificial whiskers, such as the development of thin films of conductive nanomaterials, such as nanotubes, nanowires, nanoflakes, or nanoparticles, and embedding them into elastic polymers.^[^ ^15^, ^16^, ^26^ ^]^ These approaches have the advantages of high sensitivity and ease of fabrication. However, sensitive artificial whiskers based on conductive nanoparticles have limited reliability and active operation range due to irreversible breakage between the nanoparticles when the substrate is bent or stretched to the extreme. In addition, to the best of our knowledge, previous studies focused on the development of whiskers did not provide an integrated platform where both e‐skin and whiskers can be sensitive to mechanical stimuli. Fabrication of integrated flexible e‐skin comprising artificial whiskers that can be applicable for flexible electronics is still a great challenge. In addition, achieving a simpler sensor design that effectively decouples additional external stimuli is imperative. While current soft multifunctional sensors can successfully isolate two input signals, achieving discriminative measurements for three or more stimuli with a single sensing unit poses significant challenges. The most promising strategies for achieving this include developing innovative sensing mechanisms with distinct responses to specific stimuli and employing advanced machine learning to thoroughly analyze the intertwined signals.^[^ ^23^ ^]^ Therefore, the development of a single flexible sensor unit comprising both sensitive skin and sensitive whiskers, which can discriminate coupled input signals, is of great importance.

In this study, we developed the first approach of fabricating a flexible artificial skin integrating sensory active units, including e‐skin and an artificial whisker, by embedding carboxylic acid‐functionalized multiwalled carbon nanotube (COOH‐MWCNT)/silver nanowire (AgNW)‐coated leaf skeleton and vein into a reverse microhoodoo‐patterned polydimethylsiloxane (PDMS) thin film. The developed artificial skin mimics both the design and sensory performance of the rodents’ biological skin. The artificial skin with active sensing skin and whiskers offers a comprehensive improvement over existing flexible sensors by providing active dynamic sensory functions for subtle and vigorous stimuli, multimodal sensing capabilities, improved autonomy and precision, and bioinspired design features. Moreover, in contrast to existing systems, the developed artificial whisker can offer directional sensing capabilities, allowing the system to determine and analyze the direction of stimuli like airflow, which can be crucial for specified applications in robotics and prosthetics. In this integrated platform, both the whisker and skin can precisely sense the applied mechanical stimuli, including airstreams and subtle/vigorous touch. While we didn't utilize multifunctional sensors, we employed a singular sensing unit to discern between various coupled stimuli, uncovering novel applications in the process. As proof of concept, we incorporated the developed artificial skin into a radio‐controlled robotic rodent to detect mechanical stimuli and obstacles. Using this platform, the robotic rodent can detect surrounding obstacles and airstreams as well as navigate through obstacles and channels, which is promising for tactile sensing and blind perception. Further, the robotic rodent can discriminate between three different stimuli including skin touch, whisker touch, and whisker airstream sensing.

## 2 Results and Discussion

### 2.1 Artificial Skin Structures

**Figure 1:**
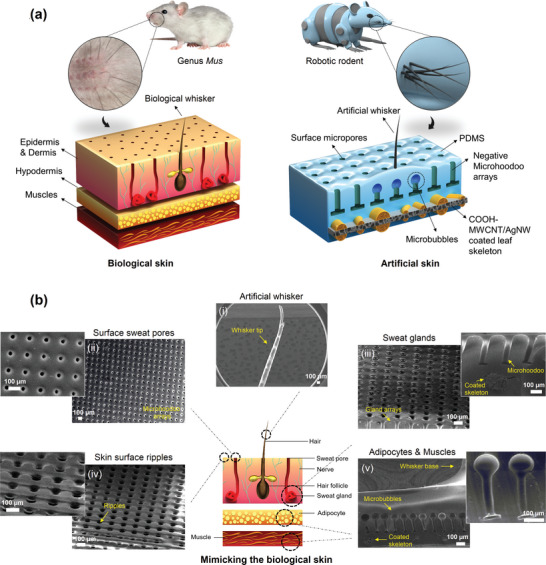
The structure of the artificial skin. a) Resemblance of biological skin to the developed artificial skin. b) FESEM images of the artificial skin structures and their similarity to biological counterparts. The structural design and physical characteristics of the artificial skin comprise negative microhoodoo structures mimicking the biological skin and whisker of rodents, including the epidermis, dermis, hypodermis, and muscles; i) COOH‐MWCNT/AgNW‐coated artificial whisker, ii) artificial skin surface pores, iii) artificial skin negative microhoodoo‐based microstructures, iv) surface ripples generated by negative microstructures, v) uniform formation of congruent microbubbles beneath the artificial whisker.

**Figure** **1**a demonstrates the schematics of biological skin and developed artificial skin. The main biological skin structures include the whisker, epidermis, dermis, hypodermis, and muscle, which provide active sensation of applied mechanical stimuli. Rodents possess highly sophisticated sensing capabilities in their skin and whiskers, which are essential for their survival and interaction with the environment. Their skin, particularly the epidermis and dermis, is equipped with a variety of mechanoreceptors that detect pressure, touch, and vibrations, allowing rats to respond to a range of environmental stimuli. The whiskers, or vibrissae, are even more specialized. Each whisker is embedded in a follicle richly supplied with nerve endings, enabling the detection of subtle changes in airflow and vibrations. This allows rats to sense nearby objects and navigate through dark or confined spaces with remarkable precision. The integration of skin and whisker sensory inputs provides rats with a comprehensive understanding of their surroundings, enhancing their ability to forage, avoid predators, and engage in social interactions. Inspired by the unique sensory characteristics of rodents, we developed an integrated, flexible artificial skin composed of active electronic skin and an artificial whisker, capable of measuring and discriminating between subtle and vigorous stimuli.

In our artificial skin, to create electrodes that are both highly conductive and flexible, the leaf skeleton was utilized due to its fractal‐like connections between veins and spongy structures on a microscale. By coating the leaf skeleton with an optimized mixture of COOH‐MWCNTs and AgNWs, flexible electrodes were fabricated. An artificial skin device was designed with a leaf‐based whisker implanted on the coated leaf skeleton electrodes embedded in a PDMS thin film (Figure 1a). The COOH‐MWCNT/AgNW‐coated leaf skeleton demonstrated a fractal network‐like microstructure that provided high surface coverage, stability, and efficient thermal and electrical distribution. Cross‐sectional images of the coated leaf skeleton demonstrated a spongy mesophyll structure (Figure S1, Supporting Information) with various surface breaches that allowed the COOH‐MWCNT/AgNW to penetrate, which improved thermal conductivity, electrical conductivity, and mechanical robustness. Each microstructure of the artificial skin was designed to provide the highest degree of resemblance to the rodents' biological skin and whisker.^[^ ^35^ ^]^ The rat's whisker with a base diameter of ≈50–175 µm,^[^ ^36^, ^37^, ^38^ ^]^ includes a surface texture composed of semiregular scales with an internal structure composed of cuticula, cortex, and void‐like medullar channel components.^[^ ^39^ ^]^ These characteristics are similar to those of the developed porous COOH‐MWCNT/AgNW‐coated leaf‐based whisker with a diameter of 90–180 µm and longitudinal internal microchannel structure (Figures S1–S3, Supporting Information). In this regard, the coated leaf skeleton provides a resemblance to the muscular system owing to its porous structure, consisting of tightly packed fibers and the extensive extracellular space filled with fibrils.^[^ ^40^ ^]^ From the top, the COOH‐MWCNT/AgNW‐coated leaf vein shows a resemblance to the biological hair and transfers the mechanical stimulation to the lower sensing layers of the artificial skin (Figure 1b (i)).

All structural characteristics of biological skin are developed in the artificial skin, including the epidermis, dermis, muscles, and hypodermis. The skin model designed in this study exhibits the biological characteristics of rodent skin, extending from paw to face. *Epidermis*. Figure 1b (ii,iv) shows the surface and texture of the developed artificial skin, which comprises uniform micropatterned negative microhoodoo re‐entrants and semi‐rippled surfaces that imitate the structure and presence of surface pores and biological rodent skin surface textures. Surface pores include both sweat pores and hair follicles. The rippled and porous surfaces provide enhanced contact with external surfaces, thereby facilitating higher sensitivity in the developed artificial skin than the flat surface skin.^[^ ^15^, ^16^ ^]^ *Dermis*. Figure 1b (iii,v) shows the structure of the developed skin that imitates the dermis, which includes negative microhoodoo patterns that imitate the sweat and whisker glands. The negative microhoodoo patterns generate rippled surfaces, surface pores, and engineered internal microstructures for the formation of microbubbles, which play a crucial role in enhancing the tactile sensitivity of the e‐skin and whisker. A rippled surface improves pressure sensitivity by increasing surface area, enabling localized deformation, concentrating stress, and enhancing contact mechanics. In addition, the presence of negative re‐entrant microstructures facilitates the formation of uniform vertical air gaps and voids through the dielectric layer, which enhances the deformability and tactile sensitivity of the e‐skin.^[^ ^21^ ^]^ *Hypodermis*. Figure 1b (v) shows the uniform formation of congruent spherical air pockets under the whisker implantation region, which imitates the biological hypodermis adipocytes. The uniform microbubbles within the artificial skin deform under applied tension, thereby changing the effective dielectric constant and compressive modulus and resulting in enhanced sensing capabilities. *Muscles*. The COOH‐MWCNT/AgNW‐coated leaf skeleton shows a porous substance comprising closely arranged fibers and fibrils similar to those of biological muscle (Figure 1b (iii,v)). Therefore, all aspects of the developed artificial skin are designed to imitate the structural characteristics and sensing functions of biological skin.

### 2.2 Mechanism of Sensitivity and Physical Characteristics

**Figure 2:**
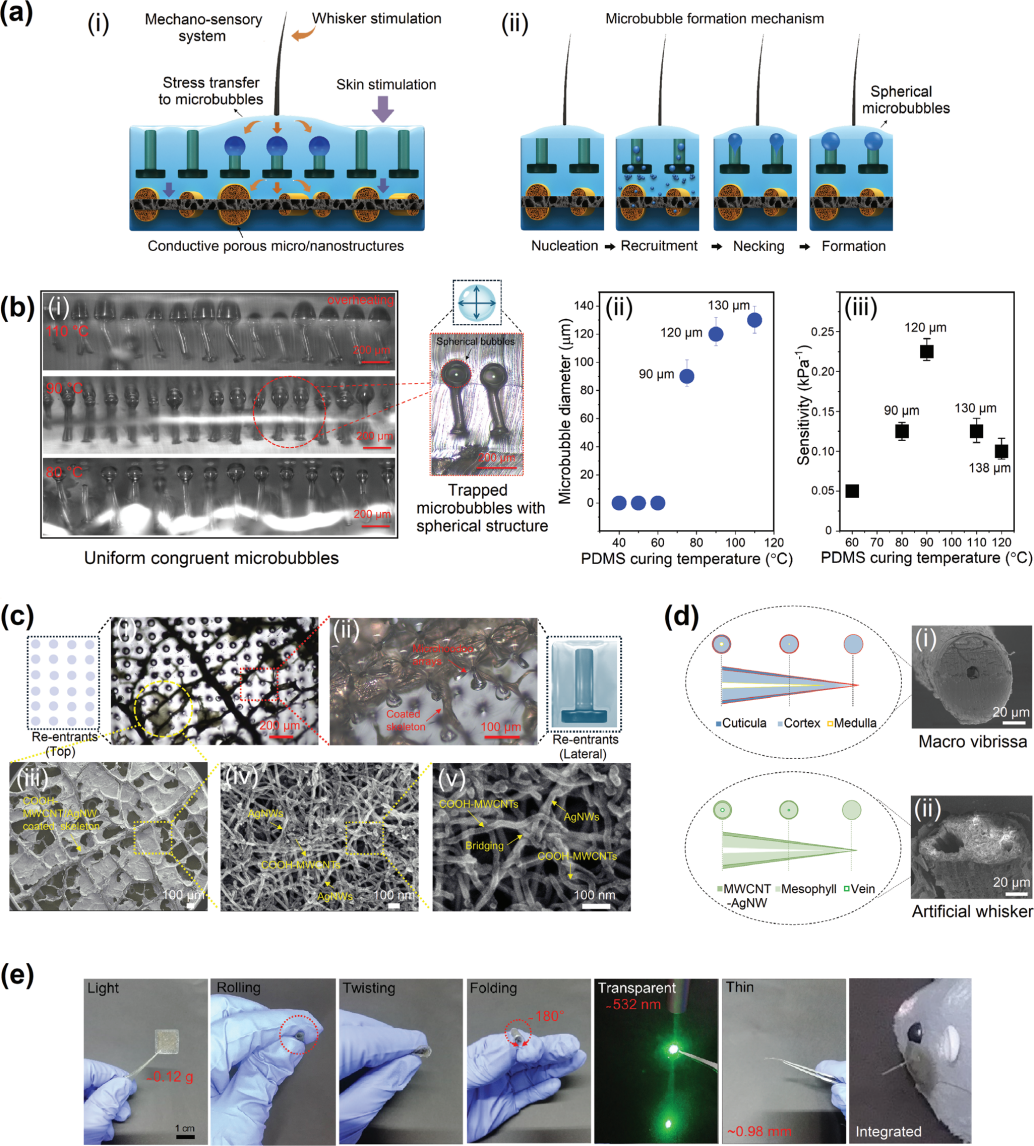
Mechanism and physical characteristics of the artificial skin. a) Mechanism of mechano‐sensory perception and microbubble formation in the artificial skin. b) Influence of PDMS curing temperature on the formation of microbubbles, microbubble diameter, and artificial skin sensitivity. c) Structure and formation of AgNWs and COOH‐MWCNTs on the leaf skeletons. d) Comparison of rat's whisker “macro vibrissa” with the developed artificial whisker at the base. SEM image of macro vibrissa, Copyright, 2011, Danja Voges.^[^ ^39^ ^]^ e) Flexibility and physical characteristics of the artificial skin.

The conductive leaf skeleton‐based active layer has a highly porous architecture comprising numerous aligned microfibers and air pockets. These microstructures deform under mechanical whisker or skin stimulation translated from the top layers, resulting in a capacitance change and rapid response (**Figure** **2**a (i)). In this study, sensitivity was enhanced by modifying the dielectric materials via the inclusion of air gaps or augmenting their dielectric constants. The addition of air gaps, whether on the surface or inside the dielectric material, results in a reduction of the effective compressive modulus.^[^ ^41^, ^42^ ^]^ Further, it enables an increase in the effective dielectric constant when compressed, as the proportion of air is substituted with solids that possess higher dielectric constants. The enhanced sensitivity of the developed artificial skin is attributable to two main factors: 1) there is far less elastic resistance in the negative microhoodoo‐patterned PDMS film because of the presence of air bubbles and 2) when patterned PDMS film is compressed, the displaced volume is air, which has a lower dielectric constant (ɛ = 1.0) than PDMS (ɛ ≈3.0), thereby increasing the effective dielectric constant and capacitance. The effective compressive modulus and dielectric constant of a composite material containing air bubbles can be estimated using certain models and equations.^[^ ^42^ ^]^ The presence of air bubbles in prepared PDMS film can potentially reduce the effective compressive modulus of the artificial skin. The effective compressive modulus represents the material's resistance to compression and is influenced by the properties of both the matrix material (in this case, PDMS) and any inclusions or voids within it. When air bubbles are introduced into the PDMS matrix, they create regions of lower stiffness and density compared to the PDMS itself. As a result, these air‐filled regions can contribute to a reduction in the overall effective compressive modulus of the composite material. This is because the compressive modulus of air is much lower than that of PDMS. For a composite material with air bubbles, one common model is the rule of mixtures.^[^ ^43^ ^]^ The rule of mixtures for the effective bulk modulus (*K_eff_*) can be expressed as:

(1) $$K_{eff}=\Phi \cdot K_{air}+\left(1-\Phi \right)\cdot K_{matrix}$$

where *Φ* is the volume fraction of air (volume of air/volume of composite), *K_air_* is the bulk modulus of air, and *K_matrix_* is the bulk modulus of the matrix material. Equation (1) shows as the volume fraction of air (*Φ*) increases, the term *Φ·K_air_* becomes a more dominant factor in the equation. Since the bulk modulus of air *K_air_* is much lower than the bulk modulus of the matrix material (PDMS) (*K_matrix_*), an increase in the contribution of the air phase (*Φ·K_air_*) results in a larger impact on the overall effective bulk modulus. The term (1‐*Φ*)·*K_matrix_* represents the contribution of the matrix material to the overall bulk modulus. Since *Φ* is increasing, the contribution of the matrix material decreases proportionally. The net effect is that the more air is introduced (increase *Φ*) the more the overall effective bulk modulus is influenced by the lower bulk modulus of air, leading to a decrease in the effective bulk modulus of the composite material.

For the effective dielectric constant (*ɛ_eff_*) of a composite with air bubbles, another common model is the Maxwell‐Garnett model,^[^ ^44^, ^45^ ^]^ which is given by Equation (2):

(2) $$\varepsilon_{eff}=\varepsilon_{matrix}\frac{1+2\cdot \chi }{1-\chi }$$

where *ɛ_matrix_* is the dielectric constant of the matrix material, and *χ* is the volume fraction of air (volume of air/volume of composite). An increase in the volume fraction of air increases the influence of the lower dielectric constant of air on the overall effective dielectric constant (*ɛ_eff_*). The denominator in equation (1*−χ*) is affected by the decrease in *χ*; however, because *ɛ_matrix_* is typically larger than 1, the effect of 1−*χ* is less pronounced. The net effect is that the more air is introduced (increase in χ), the more the overall effective dielectric constant is influenced by the lower dielectric constant of air, thereby increasing the effective dielectric constant of the composite material and capacitance (see Note S1, Supporting Information). In summary, according to the Maxwell–Garnett model, an increase in the volume fraction of air (or the presence of more air bubbles) increases the effective dielectric constant of the composite material because the lower dielectric constant of air contributes more significantly to the overall effective dielectric constant. Therefore, the presence of uniform spherical air pockets enhances the sensitivity and capacitance response of the artificial skin. Figure 2a (ii) shows the mechanism behind the formation of spherical air pockets in second‐stage heating can be explained by the formation of cavities in the non‐degassed PDMS and the recruitment of trapped air inside the coated leaf skeleton. The formation of cavities occurs exclusively during high‐temperature curing (80–90 °C) of nondegassed PDMS, indicating that the development of cavities likely starts with the enlargement of gas confined within the negative micropatterned arrays, thereby inducing the displacement of any viscous prepolymer that may have infiltrated. This process also triggers the transmission of residual air from the prepolymer to the affected area. Expanding the gas requires a temperature above room temperature; the absence of cavity formation during curing at room temperature confirms this. This observation aligns with findings from an earlier investigation that reported the formation of gas bubbles when fluid flows over a heated substrate designed with cavities.^[^ ^46^ ^]^ Overall, the mechanism behind this phenomenon comprises three stages: a) thermally induced expansion of trapped gas in microhoodoo arrays (nucleation), b) recruitment and accumulation of trapped air from porous coated leaf skeletons (growth), and c) increase in gas–liquid interface surface tension and formation of spherical cavities (necking & formation). The negative microhoodoo arrays facilitate the formation of surface pores and generate internal microbubbles within the e‐skin. Figures 2b (i, ii) show the effect of the PDMS curing temperature on the physical characteristics of microbubbles and the performance of the developed artificial skin. According to the Griffith–Willis study, the radius of bubble curvature can be efficiently engineered by controlling the temperature, and there is a direct relationship between sample temperature and spherical air pocket diameter.^[^ ^46^ ^]^ The 90 °C heating at the second stage led to the formation of uniform air pockets inside the PDMS layer above the negative microhoodoo arrays. Only when non‐degassed PDMS is cured at a high temperature (80–90 °C) do microbubbles develop. This indicates that the expansion of trapped gas inside the negative micro‐patterned arrays, which expels any viscous prepolymer that may have crept inside, is likely the first step in microbubble development. Additionally, this starts to recruit any leftover prepolymer air to the site. Moreover, there is a direct relationship between negative microhoodoo wall temperature and microbubble diameter, and with the increase of temperature from 60 to 110 °C, the diameter size increased significantly. However, the diameter reached almost saturation after 110 °C (Figure 2b (ii)). With the increase of microbubble diameter, we expect to produce higher sensitivity e‐skin. However, Figure 2b (iii) shows that, after 90 °C, the increased diameter of the e‐skin cannot provide higher e‐skin sensitivity. This phenomenon can be explained by the effect of temperature on the COOH‐MWCNT/AgNW‐coated leaf skeleton, where AgNWs oxidized and snapped under the influence of high temperatures, thereby increasing electrical resistance (Figures S4 and S5, Supporting Information) and decreasing e‐skin sensitivity at high temperatures. As demonstrated in Figures S4 and S5 (Supporting Information), the electrode's sheet resistance remains stable in the temperature range of 0–110 °C. Beyond 110 °C, a rapid increase in sheet resistance was observed due to oxidation and snapping of the AgNWs. Therefore, within the typical operating range of 0–110 °C, the system's performance remains unaffected by temperature variations, and the impact of external temperature on the flexible artificial skin can be considered negligible.

The electrical conductivity of the leaf electrode is reliant on the quantity of MWCNTs loaded into the AgNW network. The sheet resistance of the coated leaf electrode reduces significantly with an increase in COOH‐MWCNTs loading, in agreement with previous findings.^[^ ^47^ ^]^ This could be due to the flexible COOH‐MWCNTs acting as bridge connectors between AgNWs, which promotes contact between AgNWs and enhances electron transfer pathways through the AgNW network (Figures 2c (i–v)). However, the sheet resistance increases again beyond a certain COOH‐MWCNT concentration (5 wt%), as the length of AgNWs (≈25 µm) is significantly greater than that of COOH‐MWCNTs (≈1 µm) (Figure S6, Supporting Information). These results are attributed to the superior electron transfer capacity of the AgNWs compared with that of COOH‐MWCNTs. Beyond the optimal concentration, a decrease in AgNW concentration reduces electron transfer pathways, ultimately decreasing the overall electrical conductivity of the COOH‐MWCNT/AgNW network. Based on these observations, a concentration of 5 wt% was selected as the optimum level to produce an electrode with a low sheet resistance of 0.58 Ωsq^−1^. The stable interaction between COOH‐MWCNTs and AgNWs was confirmed by the bending, deforming, and wrapping of COOH‐MWCNTs within the AgNW network (Figure 2c (v)), which is supported by molecular dynamic simulation studies and improves electrical and thermal conductivities in the AgNW‐based electrodes (Note S2, Supporting Information).^[^ ^47^, ^48^, ^49^ ^]^

Figure 2d compares the structures of a rat's whisker (Figure 2d (i)) with the developed COOH‐MWCNT/AgNW‐coated artificial whisker (Figure 2d (ii)), which demonstrates the structural resemblance between the two counterparts. A hollow structure inside the artificial whisker reduces the overall mass of the whisker without significantly compromising its structural integrity. This reduction in mass makes the whisker more responsive to external forces, allowing for greater flexibility. In addition, the hollow design lowers the moment of inertia, which means the whisker can bend more easily with less force. This is particularly important for applications requiring delicate touch or sensitivity to subtle forces.^[^ ^31^, ^32^ ^]^ Moreover, the stiffness of a whisker is a function of its cross‐sectional geometry. A hollow structure can provide an optimal balance between stiffness and flexibility, ensuring that the whisker can bend without breaking. Also, hollow structures can improve the damping characteristics of the whisker, which is the ability to dissipate energy from vibrations or impacts.^[^ ^31^, ^32^ ^]^ This can enhance the whisker's ability to detect and respond to mechanical stimuli. Figure 2e shows the physical characteristics of the artificial skin, which shows the lightweight, high flexibility, favorable transparency (Figure S7, Supporting Information), and low thickness of the developed e‐skin. These characteristics contributed to the performance enhancement of the developed artificial skin for practical applications. In addition, the COOH‐MWCNT/AgNW‐coated leaf skeleton electrodes stably maintained electrical characteristics for ≈1 month under exposure to ambient temperature and humidity (Figure S8, Supporting Information). Although the sensor's capacitance and sheet resistance exhibit slight changes over time, these changes are relatively small (e.g., 0–20 days, resistance 4.0–4.4 Ω, respectively). The exponential increase in resistance may suggest a gradual degradation process, but the overall impact on performance remains minimal within the observed timeframe. The sensor's electrical characteristics were still considered stable for ≈1 month under ambient conditions.

### 2.3 Artificial Skin Sensing Performance and Blind Perception

#### 2.3.1 E‐Skin Sensing Performance

**Figure 3:**
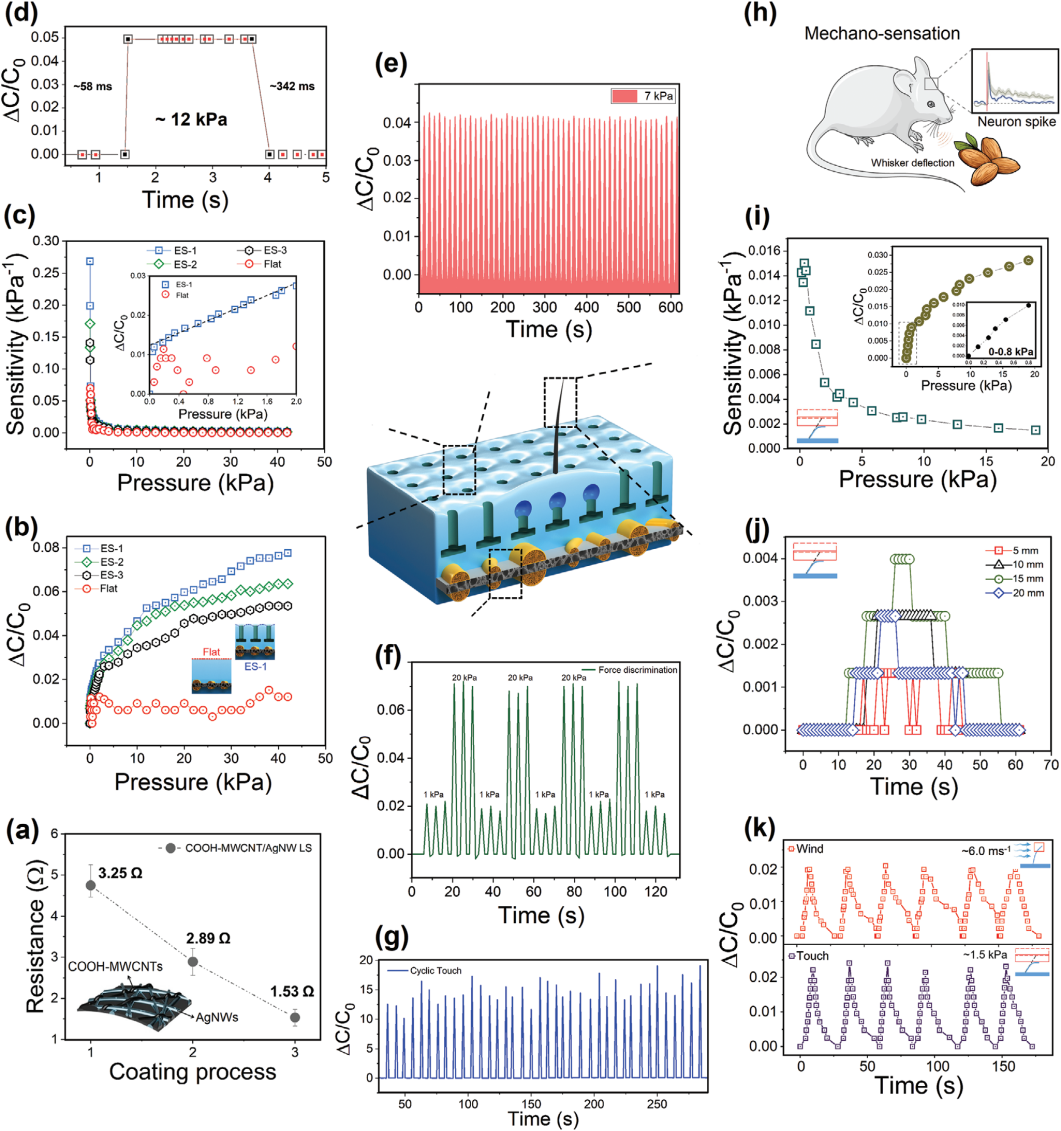
Sensing the performance of the artificial skin. a) Resistance changes of COOH‐MWCNT/AgNW‐coated leaf skeleton electrodes for the different number of the coating process (frequency, measured by LCR meter). b) Capacitive response of the artificial capacitive skins at different pressures for different samples including flat and samples ES‐1, ES‐2, and ES‐3 with CTC of 140, 160, and 200 µm, respectively. c) Sensitivity measurements and linear range of the artificial capacitive skins at different external pressures. The inset demonstrates the sensing linearity range of ES‐1 and flat samples. d) Reaction and response time of the developed artificial skin over 12 kPa applied pressure. e) Cyclic 7 kPa pressure sensing capability. f) Subtle and vigorous force discrimination capabilities of the artificial skin. g) Performance for the vigorous cyclic touch on the artificial skin. h) Mechano‐sensory function of a rodent. The inset graph shows the natural response of the biological whisker to the mechanical stimuli. i) Capacitive response of an artificial whisker at different applied pressures. Insets are the sensitivity and linearity of an artificial whisker. j) Impact of whisker length on artificial skin response to mechanical stimulation. k) Mechano‐sensory response of the whisker to the applied mechanical ≈1.5 kPa and wind stimulation, ≈6.0 ms^−1^.

The frequency of the coating process is an essential parameter in the improvement of electrical properties of COOH‐MWCNT/AgNW‐coated leaf skeletons. **Figure** **3**a shows that with an increase in the number of coatings to three times, the active layer resistance decreased from 3.25 to 1.53 Ω. However, increasing the number of coatings to four does not result in a significant reduction in sheet resistance compared to thrice coatings. This suggests that the majority of conductivity improvements are achieved with the first few coatings. Figure S9 (Supporting Information) shows the conductivity improvement reaches a plateau with three coatings, as further coatings yield minimal additional benefits. In addition, increasing the number of coatings adds to the thickness of the electrode, which can reduce its flexibility and may impact the overall performance of the device. Additionally, more coatings increase the material usage and cost, making the process less efficient. Therefore, the thrice‐coated COOH‐MWCNT/AgNW leaf skeletons were chosen to fabricate the active layer. Sensitivity measurements and a force gauge were performed on the fabricated artificial skin, comprising a COOH‐MWCNT/AgNW‐coated leaf skeleton embedded in negative microhoodoo‐patterned PDMS across a pressure range of 0.01–42 kPa, which covers the typical pressure range of daily human activities.^[^ ^50^ ^]^ The artificial skin was subjected to a stepwise increase in pressure up to 42 kPa, with a constant speed, while being closely monitored and controlled by a force gauge unit. The capacitance and e‐skin sensitivity were then measured using Equation S1 (Supporting Information), where P denotes the applied pressure and ΔC denotes the change in capacitance (C−C_0_), as outlined in Note S1 (Supporting Information).^[^ ^51^ ^]^ Figures 3b,c, and Figure S10 (Supporting Information) show the capacitance variations and sensitivity of the artificial skin over a wide pressure range for three samples with varied negative microhoodoo patterns, each having different center‐to‐center (CTC) range characteristics, including samples ES‐1, ES‐2, and ES‐3, with CTC values of 140, 160, and 200 µm, respectively, along with a flat surface structure. The micropatterned e‐skin exhibited a superior capacitive response to pressure loading compared to the flat e‐skin. Among the micropatterned samples, ES‐1 demonstrated the highest response because it contained a higher number of re‐entrant microstructures per area compared to ES‐2 and ES‐3. Therefore, the ES‐1 sample was chosen as the final sample for complementary experiments and applications. At low‐pressure range loading (<1 kPa), a maximum sensitivity value of 0.27 kP^−1^ was observed for ES‐1, which is favorably comparable to the sensitivity values achieved by existing biodegradable‐ and synthetic polymer‐based capacitive sensors (Table S1, Supporting Information).^[^ ^15^ ^]^ In addition, the micropatterned e‐skin sensitivity is higher (0.27 ± 0.09 kPa⁻1) than that of the flat e‐skin (0.08 ± 0.03 kPa⁻1), confirming the importance and impact of surface ripples in the negative micropatterned e‐skin in increasing surface contact and enhancing pressure sensitivity (Figure 3c). In addition, the micropatterned e‐skin ES‐1 exhibited a superior linear response (0–2 kPa) compared to the flat e‐skin (0–0.2 kPa) (Figure 3c, inset).

The dissimilarity in linearity between the flat and micropatterned e‐skins can be explained by the contact area variation, which is directly proportional to the electric current. The Archard model indicates that under normal pressure, a micropatterned structure comprising surface ripples experiences an almost linear increase (power of 0.89) in the contact area as the compressive load is applied.^[^ ^17^, ^52^, ^53^ ^]^ Conversely, the Hertz model predicts that for flat structures with smooth surfaces, the contact area increases with applied pressure only to a power of 0.67.^[^ ^17^, ^52^, ^53^ ^]^ In the COOH‐MWCNT/AgNW‐coated leaf skeleton embedded in negative microhoodoo PDMS patterns, an increase in the applied pressure leads to a rise in the number of small protuberances and ripples that are in contact. Additionally, according to the Hertz model, each contacting protuberance undergoes individual deformation. Consequently, an almost linear relationship between the total contact area and applied pressure ensues because of the increase in the number of deformable small protuberances and the contact area of each protuberance (Note S3, Supporting Information). Further, the patterned artificial skin exhibited a low limit of detection of 20 Pa. In addition, the developed artificial skin exhibited a rapid response (58 ms) and relaxation time (342 ms) under applied mechanical pressure of ≈12 kPa (Figure 3d). Figure 3e presents the sensing capabilities of the artificial skin in detecting the cyclic 7 kPa applied pressures. This capacitive e‐skin can conform to the contours of the human body, providing real‐time monitoring and detection of physiological signals and joint motions in an efficient, noninvasive, and user‐friendly manner. Overall, the integrated artificial skin demonstrates sensitive performance in detecting various mechanical forces and discriminating between subtle and vigorous forces (Figures 3f,g and Video S1, Supporting Information).

#### 2.3.2 Whisker Sensing Performance

Tactile sensory input from whiskers offers nocturnal rodents, such as mice and rats, crucial textural and spatial details about their surrounding environment (Figure 3h). The intricate process, including the generation of neural spikes due to the mechanical deflection of whiskers, allows rats to efficiently convert mechanical stimuli from their whiskers into meaningful neural signals, enabling them to interact with and interpret their environment effectively. To study the sensing performance of the whisker in response to different stimuli, such as touch, obstacles, and airstreams, various experiments were performed. Figure 3i shows the capacitive response, sensitivity, and linear response of the artificial whisker to the applied pressures (0–20 kPa). The whisker exhibited a tactile sensitivity of 0.015 ± 0.009 kPa^−1^ and a linear response in the range of 0–0.8 kPa. The performance of the whisker at low applied pressures exhibits linearity, akin to the linearity of the e‐skin, thus confirming the reliability and seamless integration of the artificial whisker into the e‐skin. This integration facilitates the highly sensitive response of artificial skin to a diverse array of applied stimulations, encompassing both subtle and vigorous stimuli. To further understand the impact of whisker length on sensing performance, various whisker‐implanted skins with different whisker lengths (5, 10, 15, and 20 mm) were fabricated. Figure 3j shows that the 15 mm whisker has the highest response. The 10 and 20 mm whiskers exhibited similar sensitivities to the mechanical stress (1.5 kPa). However, the 10 mm whisker exhibited a longer response time and shorter relaxation time than the 15 and 20 mm whiskers. The 5 mm whisker response is weaker than that of the other whiskers. In general, longer whiskers are more sensitive. Longer whiskers are often more flexible and have a larger range of motion. This increased flexibility allows longer whiskers to bend more feasibly in response to subtle changes in the environment, enhancing their ability to detect various mechanical forces. In addition, longer whiskers can cover a larger spatial area, allowing the sensory system to sample a broader range of environments. This increased spatial resolution enables the detection of finer details and variations in the applied stimuli. Longer whiskers provide a longer lever arm, meaning that any mechanical force applied at the whisker tip produces a larger displacement at the base, where the whiskers transfer the applied forces to the embedded microbubble networks. These mechanical advantages amplify the sensory input, making longer whiskers more sensitive to subtle changes. However, whiskers longer than the optimal length may become stiffer and less flexible with more mass and greater inertia. This increased mass can generate limitations for the whisker to transfer the applied force quickly in response to the applied force variation, which increases the response and relaxation time of the 15 and 20 mm whiskers compared with those of the 10‐mm whisker. Rapid responses are crucial for detecting dynamic stimuli and effectively navigating the surroundings. In addition, longer whiskers are more susceptible to breakage, especially in environments with obstacles or tight spaces. The risk of breakage could limit their effectiveness in providing continuous and reliable sensory information. Thus, adjusting the optimal length is essential for the effective performance of the integrated artificial skin. Choosing 10 mm as the optimal whisker length can balance sensitivity, flexibility, and practical considerations for field applications. Therefore, because of its more uniform response and relaxation and better mechanical stability, the 10 mm whisker was chosen for further experiments. Figure 3k shows the impact of cyclic mechanical force and wind on the 10 mm whisker, which shows a reversible sensing performance. The implanted whisker demonstrated a sensitive response to the mechanical stimulation, and there was a high similarity in response to stimulation loading and unloading (Figure S11, Supporting Information), confirming the whisker's sensitive performance. In addition, the whisker‐implanted skin detected cyclic wind loading/unloading (Figure S12, Video S2, Supporting Information). Therefore, the developed integrated artificial skin can be used to detect both mechanical and wind stimuli.

### 2.4 Obstacle Sensing and Blind Perception by an Incorporated Robotic Rodent

**Figure 4:**
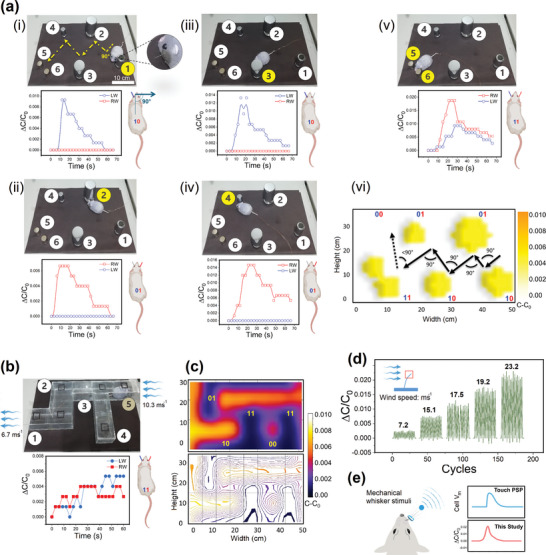
Tactile sensing and blind perception by the artificial skin‐incorporated robotic rodent. a) (i–v) Rodent movements on the stage composed of six different obstacles and the capacitive response of right and left whiskers to the obstacle stimulation. (vi) Approximate surface map of the stage board for the constant movement of the rodent on the board including the translation of capacitive response to the binary language and the navigational commands to turn and move through the obstacles. b) Tactile sensing and blind perception by the artificial skin‐incorporated robotic rodent in the escape cell under constantly applied wind. Rodent movements on the glass escape cell composed of open and dead‐end channels and the capacitive response of right and left whiskers to the wind stimulation. Plot shows the mechanosensory response for position 5. c) Capacitance response map and contour map of the escape cell for rodent motions on the multi‐channel cell including the translation of capacitive response to the binary signals and to the navigational commands to move through the channels. d) Response of the artificial whisker to the wind with different speeds. e) Comparison of sensory (touch) postsynaptic potentials (PSPs) in biological whiskers with the capacitive response in the developed artificial whisker.

To study the practical application of the developed artificial skin, two different experiments were designed including an obstacle stage board (**Figure** **4**a) and a wind escape cell (Figure 4b; Figure S13, Supporting Information). The obstacle stage board is composed of six cylindrical metallic obstacles with different sizes distributed ≈90° orientation to the next obstacle in the stage board (Figure 4a). The developed artificial skin connected to the LCR meter was attached to the right and left facial skin of robotic rodents. By moving the rodent in the stage in contact with each obstacle the produced signals by whisker stimulation were transmitted to the LCR meter data recording system. The data analysis can be performed by a system or rodent remote operator. The binary language of “1:0” is used for deciphering and translating the rodent response to each obstacle, in which receiving a signal is defined as 1 and lack of signal is defined as 0. The rodent navigation through the obstacle stage board is defined by receiving 1:0 responses from the left/right whiskers. By contacting with an obstacle if the rodent's remote navigation system receives a response from the left whisker defined as 1, but no response from the right whisker defined as 0, the rodent will turn right (≈90°) and move against the responsive whisker 1 toward the orientation of the non‐responsive whisker 0 to escape from that obstacle Figures 4a (i–iv). However, if both left/right whiskers engage in obstacle contact, the navigation system defines this as 1:1, which navigates the rodent to turn in angles lower than 90° and moves to escape the obstacle blockage (Figure 4a (v)). Constant movement and exploration of the stage board by the rodent provide a unique blind perception of stage board structures and architectures for remote navigation and data analysis systems (Figure 4a (vi)). The data analysis system can define the number, size, volume, and approximate shape of obstacles through the stage board, which can be used for practical applications such as remote robotic navigation and remote surface mapping.

In another experiment, a glass escape cell was designed composed of open‐ and dead‐end channels which were attached to the industrial air compressor for blowing and streaming the air ≈10.3 ms^−1^, through the entrance (point 5) and exit of the escape cell (point 1) (Figure 4b; Figures S13 (i–v), Supporting Information). The binary language 1:0 is utilized to decode and interpret the response of rodents to each section of the escape cell. When navigating through an escape cell, rodents use 1:0 responses from their left and right whiskers to navigate and escape from escape cell channels. If the rodent's navigation system receives a response from the right whisker defined as 1 upon stimulation by the wind, but no response from the left whisker, defined as 0, the rodent will turn and move ≈90° toward the responsive whisker 1 against the orientation of the non‐responsive whisker 0, in contrast to the previous experiment, to escape from the channels (Figure S13 (ii), Supporting Information). However, if both left and right whiskers feel the wind, the navigation system defines this as 1:1, which causes the rodent to turn and move at angles bigger than 90° to avoid obstacle blockage (Figures S13 (i,iii,v), Supporting Information). If none of the whiskers feels the wind 0:0 (Figure S13 (iv), Supporting Information), the rodent will turn and move at angles less than 90° to escape the dead‐end channel.

Using the transmitted data by rodent interaction with the wind in the escape cell, the data analysis system can provide an approximate image of the escape cell and its physical characteristics. The capacitance signal intensity map and contour plot exhibit the approximate estimation of the analysis system which provides a unique tool for remote tactile sensing and blind perception using the developed platform (Figure 4c). Figure 4d shows the mechanosensory response of the artificial whisker to winds of different speeds, demonstrating the whisker's uniform performance and increased capacitive response to higher wind speeds. In Figure 4e, the mechanosensory responses of the whiskers to wind and touch reveal that the artificial whisker's response closely mimics the sensory (touch) postsynaptic potentials (PSPs) in biological whiskers,^[^ ^1^ ^]^ highlighting the biomimetic characteristics of the developed artificial whisker. The ability of artificial whiskers to generate responses that closely mimic sensory PSPs in biological whiskers is important because it enhances the functionality, accuracy, and applicability of the developed platform in a wide range of fields, from robotics and prosthetics to neuroscience and machine learning.

### 2.5 Discrimination of Skin Touch, Whisker Touch, and Wind Stimulation

**Figure 5:**
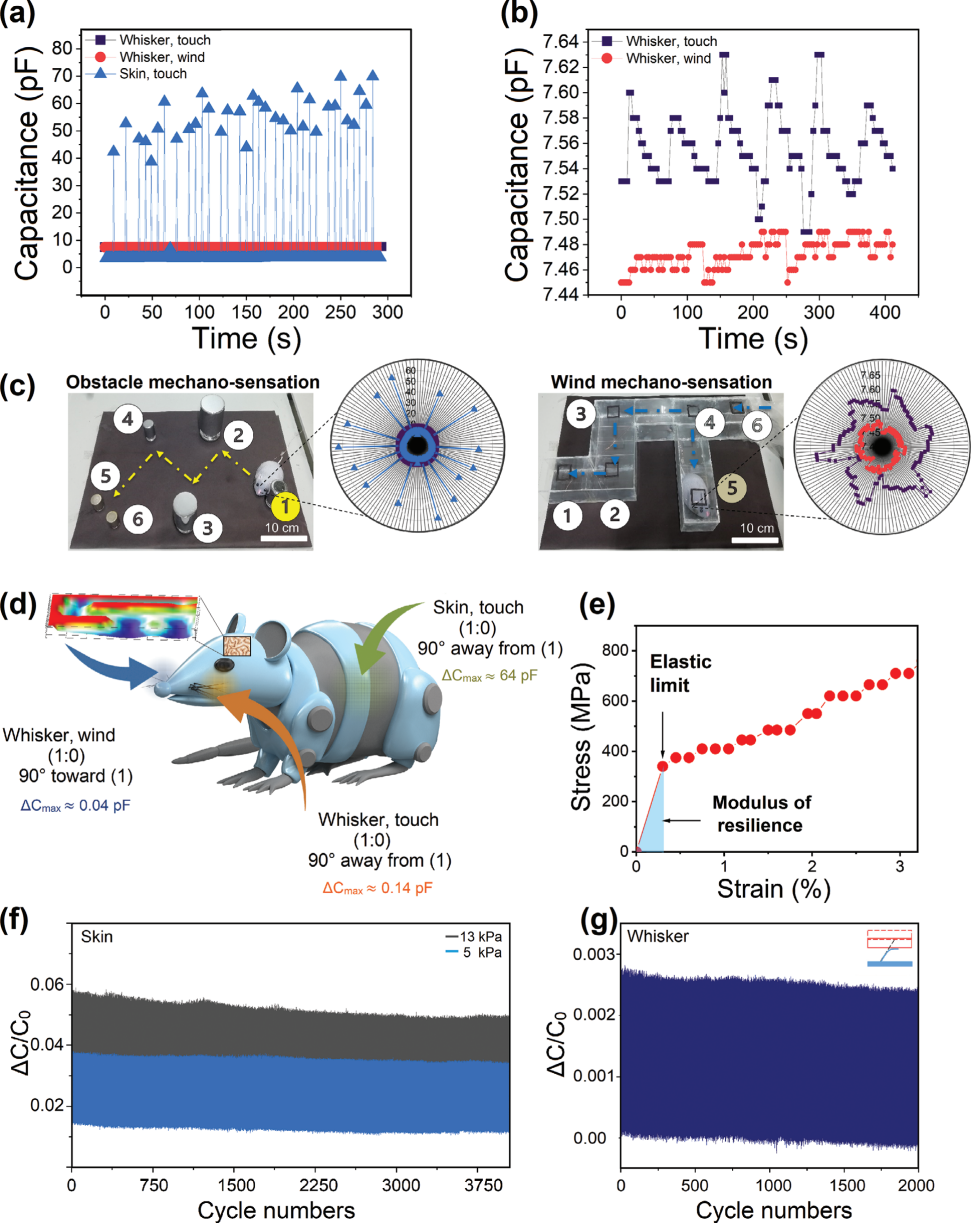
The discrimination of binary signals for blind perception and stable performance. a) The capacitance variations for e‐skin touch, whisker touch, and whisker stimulation by wind. b) The capacitance variations for whisker touch stimulation and whisker stimulation by wind. c) Radar plots for the mechano‐sensory perception of obstacles and wind by the artificial skin. d) The rodent could detect, discriminate, and decipher the produced signals by e‐skin touch, whisker touch, and wind whisker contacts based on their different capacitive responses. e) Tensile strength of developed artificial skin. f) Stability of the developed e‐skin for different cyclic pressures of 5 and 13 kPa for 4000 cycles. g) Stability of the artificial whisker for cyclic applied force (1.5 kPa) for 2000 cycles.

There are differences between binary responses for each type of stimulation. For skin and whisker touches, 1:0 means 90° turn and movement against whisker 1 direction. However, for whisker stimulation by the wind (10.3 ms^−1^), 1:0 means 90° toward whisker 1 direction. To investigate whether the rodent could decipher the binary language for each stimulation, we analyzed the capacitive response received by each type of stimulation (**Figure** **5**). Figure 5a shows the recorded capacitance for each type of stimulation during a 300 s period. The skin touch responses from 0 to 1 significantly differ (capacitance ≈4–70 pF) from the whisker stimulation. Therefore, the robotic rodent can easily understand the skin touch from the whisker stimulation. For the whisker stimulation, the touch and wind signals were in similar ranges. However, there is still a significant difference between their capacitance signals during the stimulation period. For instance, the value of 0–1 capacitance for whisker wind stimulation is ≈7.45–7.49, whereas, for whisker touch, the capacitance is ≈7.50–7.63 (Figure 5b). Figure 5c shows the radar plots for the mechano‐sensory perception of obstacles and wind by the artificial skin, indicating a significant difference between various whisker stimuli over time. The value of ΔC_max_ for skin touch is 64 pF, whereas this value is in the lower ranges of 0.14 and 0.04 pF for whisker touching and whisker wind interaction, respectively (Figure 5d). The difference in signal values can be attributed to the varying motion responses of the robotic rodent. For skin and whisker touch (1:0), a ΔC_max_ of 64 and 0.14 indicates a movement 90° away from 1. However, for whisker wind interaction, a ΔC_max_ of 0.04 indicates a movement of 90° toward 1. Therefore, based on these differences, the rodent can understand each binary signal, decipher its meaning, correspond each generated signal to the specific stimulation, and navigate through obstacles.

#### 2.5.1 Force Discrimination, Stimuli Decoupling, and Stability

The stimulus decoupling capability of the developed artificial skin refers to its ability to differentiate and independently respond to multiple stimuli, such as skin touch, whisker touch, and whisker wind interaction, even when these stimuli occur simultaneously. The artificial skin interprets binary signals, where a 1:0 response signifies different actions depending on the type of stimulation—90° movement away from whisker 1 for skin and whisker touches, and 90° movement toward whisker 1 for wind interactions. The capacitive responses differ for each type of stimulus, with skin touch showing significant changes (≈4–70 pF), whisker wind having smaller responses (≈7.45–7.49 pF), and touch interactions producing slightly higher values (≈7.50–7.63 pF) (Figures 5a,b). This differentiation is further highlighted by the maximum change in capacitance (ΔC_max_), where skin touch registers 64 pF, compared to 0.14 pF for whisker touch and 0.04 pF for wind interaction (Figure 5d). These distinctions enable the robotic rodent to interpret each binary signal correctly and navigate based on the specific stimulation. Unlike systems that rely on machine learning to process intertwined stimuli, the artificial skin's decoupling capability allows it to accurately detect and respond to different stimuli simultaneously using a single sensory unit, demonstrating its superior performance and broad potential for various applications. The developed sensor demonstrates decoupling capability through the following key mechanisms: 1) *Independent Response to Different Stimuli*: The artificial skin responds differently to various stimuli (skin touch, whisker touch, and wind interaction) despite them occurring simultaneously. It interprets binary signals where each type of stimulus produces distinct responses. 2) *Distinct Capacitive Changes*: The sensor registers different capacitance values (ΔC_max_) for each stimulus. 3) *Simultaneous Interpretation*: The sensor does not rely on machine learning or complex algorithms to interpret intertwined stimuli. Instead, its design inherently allows it to decouple signals by reading distinct capacitive responses in real time. This simultaneous interpretation ensures the sensor can handle multiple stimuli without confusion, meaning it doesn't need to untangle mixed data but rather directly separates them based on their physical impact on the sensor. The developed sensor doesn't just detect signals but discriminates between them based on distinct, measurable changes (e.g., capacitance). This ability to independently recognize and respond to simultaneous inputs is the essence of decoupling multiple signals. The fact that it uses a single sensory unit to handle multiple stimuli without mixing them is a strong indicator of its decoupling capability. **Table** **1** compares various reported artificial whiskers with our developed integrated artificial whisker, which exhibits the superior performance of the present platform in terms of the active sensory units, integration, stimuli decoupling ability, and range of applications. The capability to decouple intertwined signals of skin touch, whisker touch, and whisker wind interaction without the aid of machine learning approaches confirms the remarkable performance and potential of our integrated artificial skin for the simultaneous detection of multiple stimuli using a single sensory unit. To ensure the reliable performance and robustness of the developed e‐skin, its tensile strength and durability were analyzed (Figures 5e,f). Figure 5e confirms the mechanical robustness of the developed e‐skin with an ultimate tensile strength of ≈2000 MPa. In addition, the e‐skin demonstrated consistent and stable performance throughout the 4000 cycles under applied pressures of 5, 8, and 13 kPa (Figures 5f, S14 and S15, Supporting Information). This reliability and the ability to differentiate forces for 4000 cycles is attributable to the unique semi‐elastic porous characteristics and mechanical tensile strength (Young's modulus ≈1133 MPa) of the leaf skeleton‐based components of the e‐skin (Figures 5e; Figure S16, Supporting Information). Figure 5g shows the uniform and sensitive response of the artificial whisker to the force gauge and motion control's cyclic stimulus for 2000 cycles, which confirms the stability and robustness of the artificial whisker (Figures S17 and S18, Supporting Information). Figure S17 (Supporting Information) shows a snapshot of an artificial whisker under extreme applied force, bending up to 90° before gradually relaxing. This confirms the whisker's reversible, flexible, and durable performance. During the process, the whisker retained its structural integrity and returned to its original position after the force was removed. This highlights the efficient integration of the whisker into the PDMS‐based artificial skin. These results demonstrate the reliability, stability, unique mechanical characteristics, and robustness of the developed artificial skin for various practical applications, including wearable tactile sensors and robotic platforms.

**Table 1:** Comparison of sensing performance, characteristics, and decoupling ability of artificial whiskers with the present study.

Materials	Sensitivity	Transduction mechanism	Integration	Active skin	Active whisker	Stimuli decoupling^a^ ^]^	Ref	Application
CNT, AgNPs	≈8%/Pa,	Resistive	✗	✗	✓	✗	[26]	Gas flow monitoring
PTFE, Cu	–	Triboelectric	✗	✗	✓	✗	[27]	Obstacle avoidance and local mapping
Graphene, Polyurethane, Cu	(ΔR/R_0_)%≈1180,	Resistive	✗	✗	✓	✗	[28]	Underwater‐induced vortex detection
Ninjaflex, Conductive TPU, PI‐ETPU 95–250 Carbon Black	–	Resistive	✗	✗	✓	✗	[29]	Flexible tactile sensors
Carbon fiber, Magnet	0.38°/LSB, 0.021 Nmm/LSB,	Resistive	✗	✗	✓	✗	[30]	Angular displacement and moment sensing
Carbon fiber, Magnet, Polyimide	–	Magnetic	✗	✗	✓	✗	[33]	Multi‐directional airflow sensing
Polystyrene rod, N52 permanent magnet	–	Magnetic	✗	✗	✓	✗	[54]	Mapping of underground mining environments
PI, PDMS, Au, Cr	GF 80	Resistive	✗	✗	✓	✗	[55]	Food industry, measuring surface properties
PVDF, PDMS, Ag, CPP	0.01796 V/kPa	Piezoelectric	✗	✗	✓	✗	[56]	Vortex perceiving and underwater environmental perceiving
PDMS, PAN	0.31‐2.81 N^−1^	Triboelectric	✗	✗	✓	✗	[57]	Self‐powered obstacle detection
PDMS, CNT, AgNPs	0.0076%/ms^−1^	Piezoresistive	✗	✗	✓	✗	[58]	Diverse flow analyses
CNT, AgNP, PEDOT:PSS	59%/Pa	Resistive	✗	✗	✓	✗	[59]	3DThree‐dimensional space and temperature distribution mapping
POFETH, Silicone	0.00561 V mm^−1^	Triboelectric	✗	✗	✓	✗	[60]	Direction identification and real‐time distance sensing
Cu, SbTe_x_, Nylon, PDMS	1–20.4 m s^−1^	Thermoelectric, Piezoresistive	✗	✗	✓	✗	[61]	Tracking finger movement for gesture detection, stimuli discrimination
Present Study	skin≈∼ 0.27 27 kPa^−1^ Whisker≈∼ 0.015 kPa^−1^,	Capacitive	✓	✓	✓	✓	^−^	Tactile sensing, blind perception, obstacle detection, and avoidance, airstream detection, and navigation

^a]^ Stimuli decoupling ability without the aid of machine learning.

## 3 Conclusions

We developed an artificial skin with a highly responsive artificial whisker and an e‐skin that imitates the sensory functions of the rodent skin and whisker. The artificial whisker comprises highly flexible leaf skeleton veins coated with COOH‐MWCNTs and AgNWs to detect airstreams and navigate obstacles with high sensitivity. To enhance sensitivity, we embedded a COOH‐MWCNT/AgNW‐coated leaf skeleton in a reverse microhoodoo‐patterned PDMS thin film that imitates mammalian skin. The e‐skin contains unique, spherical, and congruent air pockets that transfer mechanical stimulation from the artificial whisker to the active e‐skin. The developed artificial skin displays high capacitance sensitivity, wide sensing range, high flexibility, low weight, and low thickness. It can detect subtle and vigorous mechanical stimuli, including airstreams and subtle touch. One of its unique features is the highly engineered integrated design that facilitates the sensitive detection, discrimination, and deciphering of different stimuli, such as skin touch, whisker touch, and whisker wind stimulation. This decoupling capability facilitates efficient object detection, blind perception, and navigation among obstacles. Thus, using the incorporated artificial skin, the robotic rodent can discriminate signals, decipher their meaning, correlate each signal to the specific stimulation, and navigate through obstacles. We believe that the unique force discrimination capability of the artificial skin will herald a new approach to the development of advanced integrated tactile sensing and blind perception platforms. The developed capacitive artificial skin has great potential for the development of biocompatible, low‐cost, sensitive, and flexible artificial skins for various applications, such as wearable electronics, HMIs, robotics, and flexible sensors.

## 4 Experimental Section

### Preparation of COOH‐MWCNT/AgNW‐Coated Leaf Skeletons Electrodes

An AgNW aqueous (0.5 wt%, deionized (DI) water) solution was purchased from C3Nano (South Korea) with an average length of 25 µm and an average diameter of 20 nm. Rubber tree leaf skeletons were obtained from “Skeleton Leaf, Just the Leaves,” United Kingdom. CTAB and gallium–indium eutectic acid (EGAIn) were purchased from Sigma–Aldrich, United States of America. COOH‐MWCNTs were purchased from US Research Nanomaterials and dispersed in DI water using a homogenizer without any dispersing agent. To fabricate electrodes, leaf skeletons were dipped in a 2 mm CTAB/DI water solution, followed by drying under ambient room conditions overnight. Positively charged leaf skeletons were then placed inside Petri dishes, along with varied concentrations of COOH‐MWCNT/AgNW solutions, and dried under ambient conditions, facilitating the soaking and absorbance of COOH‐MWCNT/AgNW on leaf skeletons.

### Fabrication of Integrated Artificial Skin

A positive microhoodoo PDMS mold was prepared using a high‐precision photolithography approach with adjusted specifications (Figures S19 and S20, Supporting Information). After removing impurities from the surface of the slide and cover glass in a 1 m sodium hydroxide solution for 1 h, the slide and cover glass were washed with distilled water. The hydroxyl groups on washed glass were activated on the surface through the O_2_ plasma treatment (Harrick Plasma PDC‐32G‐2, 30 s), and then reacted for 2 h by immersing 3‐(trimethoxysilyl)propyl acrylate in a solution diluted 5% (v/v) in ethanol. After the reaction, the glass was washed with ethanol and heat‐treated in an oven at 80 °C for 15 min. Another glass subjected to O_2_ plasma treatment was subjected to 30 µL of trichloro (1H, 1H, 2H, 2H‐perfluorooctyl) silane in a vacuum desiccator and reacted for 2 h in a vacuum state. After the silane treatment, the glass was washed with ethanol. After including the polyurethane acrylate (PUA) on the fluoroalkyl silane‐treated glass, the acrylate silane‐treated glass was covered to prevent air bubbles. At this time, glass beads were used as a spacer to secure the adjusted height of the microstructure's head. Then, the glass was exposed to the UV light in the top section to form the microstructures using inverted optical microscopy and a digital mirror device (DMD). After removing the fluoroalkyl silane‐treated glass, unreacted substances were removed using water and a small amount of ethanol. The PUA was placed on it again and the glass was covered to prevent the air bubbles. At this time, the PI tape was used as a spacer to secure the height of the microstructure's pillar. A column of the microhoodoo structure was fabricated by exposing the light smaller than the head of the microstructure to the glass. After removing the fluoroalkyl silane‐treated glass and spacer, unreacted substances were removed using water and a small amount of ethanol. Following the spin‐coating PUA on the sliding glass, the bottom of the pillar of the structure was contacted with the PUA and was cured for 30 min using UV light (365 nm, Unitec, LF‐215.L). The glass bonded to the cured head of the microstructure was removed, and the structure was treated with fluoroalkyl silane. Finally, PDMS and the curing agent were mixed at a ratio of 10:1 (w/w), poured into the structure, and cured at 80 °C for 2 h to prepare a positive microhoodoo PDMS mold. In the next step, the COOH‐MWCNT/AgNW‐coated leaf skeleton was incorporated into the mold, and the PDMS solution was cast on the entire structure. The entire system was then cured at 80 °C for 120 min. After curing, the PDMS thin film was piled, which generated negative microhoodoo patterns on the entire system, which affected the compressive moduli and dielectric constants within the e‐skin. For whisker implantation, an isolated COOH‐MWCNT/AgNW‐coated leaf skeleton vein with a specified length (5–20 mm) was used because of its high flexibility, electrical conductivity, and structural resemblance to the biological whiskers of mammals. The isolated coated vein was then implanted on top of negative microhoodoo arrays by injecting 50 µL PDMS (10:1) under optical microscopy visualization and control similar to the surgical hair implantation procedure: “follicular unit transplantation.” The entire system was then cured again at 90 °C for 45 min. The EGAIn was used for soldering, and a Cu wire was used to connect the system to the LCR meter.

### Characterization and Measurements

The structures of the leaf skeleton and dielectric layers were analyzed using field‐emission scanning electron microscopy (FE‐SEM) (JEOL JSM‐7610F, JAPAN) at the Total‐period Analysis Center for the Ulsan Chemical Industry of the Korea Basic Science Institute. Capacitance variations were measured using an LCR meter (KEYSIGHT, U1732C, USA) at a testing frequency of 1 kHz. Response and relaxation times were measured using the KEYSIGHT E4980AL precision LCR meter. To detect and measure the external pressure, a force gauge with a computer‐controlled stage (MARK‐10, series 7 (M7‐05), USA) was used. A commercially purchased radio‐controlled robotic rodent was modified by attaching the developed artificial skin to its body. Surface mapping and contour plots were prepared using Origin Pro software and generated using ImageJ with the 3D interactive surface plot plugin. The artificial skin was adhered to the robotic platform using a biodegradable ultrathin double‐sided cellulose adhesive. The impact of this adhesive on the performance of the artificial skin is evaluated by using a force gauge to measure any differences compared to scenarios without the adhesive. The observation indicated that the use of this adhesive did not significantly affect the performance of the artificial skin. The choice of the ultrathin double‐sided cellulose adhesive was based on its efficiency and neutrality, ensuring reliable attachment without altering the sensor's functionality.

Artificial skin and whiskers were subjected to forces ranging from 0 to 42 kPa, measured using a force gauge. The areas of the artificial skin subjected to the applied mechanical force were 0.5 cm × 0.5 cm for whisker sensitivity analysis and 0.7 cm × 0.7 cm for skin pressure sensitivity analysis. A custom glass escape tunnel (dimensions: 30 cm × 15 cm, with height × width: 5.2 cm × 5.2 cm) was constructed to ensure controlled wind interactions (Figure S21, Supporting Information). Wind speeds were regulated between 7.2 and 23.2 m^−1^s^−1^ using a standard air compressor, while a digital anemometer monitored the exact wind speed at the tunnel entrance. This setup aimed to minimize interference and unwanted external effects while simulating realistic environmental conditions for the robotic rodent. The artificial skin's binary signal interpretation—with responses such as “1:0” indicating specific actions like 90° movements—also contributes to its ability to decouple and respond to complex input. Thus, the flexible artificial skin's ability to decouple signals arose from the precise differentiation of capacitance changes specific to each type of stimulus. By independently processing these inputs, the system enabled the robotic rodent to interpret and navigate through obstacles without relying on machine learning algorithms or complex processing techniques. Figure S22 (Supporting Information) illustrates the approach used to measure the tactile sensitivity of the whisker. The integrated whisker was subjected to a stepwise increase in vertical force gauge pressure, up to 21 kPa, over an area of 0.5 cm × 0.5 cm at a constant speed, monitored and controlled by a force gauge unit. Simultaneously, the capacitance response of the artificial skin to whisker deflection was recorded using an LCR meter (Note S4, Supporting Information). A Nikon ECLIPSE (TI2‐S‐HU, Japan) inverted microscope was used for photolithography. Cyclic pressure experiments were performed using a five‐phase motion‐controlling device (AUTONICS, A10K). A digital anemometer (Aicevoos, AS‐H3) was used to measure the wind speed. In addition, the tensile properties were measured using a universal testing machine (ORIENTAL).

## Conflict of Interest

The authors declare no conflict of interest.

## Supporting information



Supporting Information

Supplemental Video 1

Supplemental Video 2

## Data Availability

The data that support the findings of this study are available from the corresponding author upon reasonable request.

## References

[1] C. C. Petersen , Nat. Rev. Neurosci. 2019, 20, 533. 31367018 10.1038/s41583-019-0200-y PMC7116865

[2] C. C. Petersen , Neuron 2007, 56, 339. 17964250 10.1016/j.neuron.2007.09.017

[3] R. Aronoff , F. Matyas , C. Mateo , C. Ciron , B. Schneider , C. C. Petersen , Eur. J. Neurosci. 2010, 31, 2221. 20550566 10.1111/j.1460-9568.2010.07264.x

[4] S. Hubatz , G. Hucher , D. E. Shulz , I. Férézou , Sci. Rep. 2020, 10, 763. 31964984 10.1038/s41598-020-57684-6 PMC6972923

[5] S. Crochet , C. C. Petersen , Nat. Neurosci. 2006, 9, 608. 16617340 10.1038/nn1690

[6] I. Ferezou , F. Haiss , L. J. Gentet , R. Aronoff , B. Weber , C. C. Petersen , NeuronNeuron 2007, 56, 907. 10.1016/j.neuron.2007.10.007 18054865

[7] H. Yang , S. E. Kwon , K. S. Severson , D. H. O'connor , Nat. Neurosci. 2016, 19, 127. 26642088 10.1038/nn.4183 PMC4696889

[8] S. E. Dominiak , M. A. Nashaat , K. Sehara , H. Oraby , M. E. Larkum , R. N. Sachdev , J. Neurosci. 2019, 39, 9818. 31666357 10.1523/JNEUROSCI.1809-19.2019 PMC6891063

[9] M. Lu , C. Huang , Z. Xu , Y. Yuan , M. Wang , M. Xiao , L. Zhang , P. Wan , Adv. Funct. Mater. 2023, 33, 2306591.

[10] M. Qi , R. Yang , Z. Wang , Y. Liu , Q. Zhang , B. He , K. Li , Q. Yang , L. Wei , C. Pan , Adv. Funct. Mater. 2023, 33, 2214479.

[11] G. Sun , P. Wang , Y. Jiang , H. Sun , T. Liu , G. Li , W. Yu , C. Meng , S. Guo , Nano Energy 2023, 110, 108367.

[12] X. Zhang , Z. Hu , Q. Sun , X. Liang , P. Gu , J. Huang , G. Zu , Angew. Chem. 2023, 135, e202213952. 10.1002/anie.202213952 36346155

[13] B. Nie , S. Liu , Q. Qu , Y. Zhang , M. Zhao , J. Liu , Acta Biomater. 2022, 139, 280. 34157454 10.1016/j.actbio.2021.06.018

[14] J. Chen , L. Li , Z. Zhu , Z. Luo , W. Tang , L. Wang , H. Li , Mater. Today Chem. 2022, 23, 100718.

[15] M. Zarei , G. Lee , S. G. Lee , K. Cho , Adv. Mater. 2023, 35, 2203193. 10.1002/adma.202203193 35737931

[16] G. Lee , M. Zarei , Q. Wei , Y. Zhu , S. G. Lee , Small 2022, 18, 2203491. 10.1002/smll.202203491 36047645

[17] G. Y. Bae , S. W. Pak , D. Kim , G. Lee , D. H. Kim , Y. Chung , K. Cho , Adv. Mater. 2016, 28, 5300. 27159832 10.1002/adma.201600408

[18] Y. Lee , J. Park , A. Choe , S. Cho , J. Kim , H. Ko , Adv. Funct. Mater. 2020, 30, 1904523.

[19] D. H. Ho , Q. Sun , S. Y. Kim , J. T. Han , D. H. Kim , J. H. Cho , Adv. Mater. 2016, 28, 2601. 26833961 10.1002/adma.201505739

[20] S. Lee , J. Kim , I. Yun , G. Y. Bae , D. Kim , S. Park , I.‐M. Yi , W. Moon , Y. Chung , K. Cho , Nat. Commun. 2019, 10, 1784. 31213598 10.1038/s41467-019-10465-w PMC6581939

[21] Y. Ko , C. C. Vu , J. Kim , Sensors 2021, 21, 3895. 34200047 10.3390/s21113895 PMC8200227

[22] J. Son , G. Y. Bae , S. Lee , G. Lee , S. W. Kim , D. Kim , S. Chung , K. Cho , Adv. Mater. 2021, 33, 2102740. 10.1002/adma.202102740 34396596

[23] J.‐H. Lee , K. Cho , J.‐K. Kim , Adv. Mater. 2024, 2310505.

[24] M. Zarei , J. Hoon Kim , J. Tark Han , S. G. Lee , Chem. Eng. J. 2024, 479, 147849.

[25] M. Zarei , J. H. Kim , J. T. Han , S. G. Lee , Chem. Eng. J. 2023, 470, 144306.

[26] K. Takei , Z. Yu , M. Zheng , H. Ota , T. Takahashi , A. Javey , Proc. Natl. Acad. Sci. USA 2014, 111, 1703. 24449857 10.1073/pnas.1317920111 PMC3918780

[27] P. Xu , X. Wang , S. Wang , T. Chen , J. Liu , J. Zheng , W. Li , M. Xu , J. Tao , G. Xie , Research 2021, 2021, 9864967. 38617376 10.34133/2021/9864967 PMC11014677

[28] J. Z. Gul , K. Y. Su , K. H. Choi , Soft Rob. 2018, 5, 122. 10.1089/soro.2016.0069 29297780

[29] B. Eijking , R. Sanders , G. Krijnen , in 2017 IEEE Sensors Conf., Glasgow, UK, October 2017, 1.

[30] S. Kim , C. Velez , D. K. Patel , S. Bergbreiter , in 2019 IEEE/RSJ Int. Conf. on Intelligent Robots and Systems (IROS), Macau, China, November 2019, 665.

[31] B. Mitchinson , C. J. Martin , R. A. Grant , T. J. Prescott , Adv. Robot. Res. 2004, 274, 111.

[32] M. Kaneko , N. Kanayama , T. Tsuji , IEEE Trans. Robot. Autom. 1998, 14, 278.

[33] S. Kim , R. Kubicek , A. Paris , A. Tagliabue , J. P. How , S. Bergbreiter , in 2020 IEEE/RSJ International Conference on Intelligent Robots and Systems (IROS), Las Vegas, NV, USA, October 2020, 1330.

[34] H. M. Emnett , M. M. Graff , M. J. Z. Hartmann , in Proc. of Robotics: Science and Systems, pittsburgh, pennsylvania , 2018, 10.15607/RSS.2018.XIV.059.

[35] L. W. Bosman , A. R. Houweling , C. B. Owens , N. Tanke , O. T. Shevchouk , N. Rahmati , W. H. Teunissen , C. Ju , W. Gong , S. K. Koekkoek , Front. Integr. Neurosci. 2011, 5, 53. 22065951 10.3389/fnint.2011.00053 PMC3207327

[36] C. S. Bresee , H. M. Belli , Y. Luo , M. J. Hartmann , J. Exp. Biol. 2023, 226, jeb245597. 37577985 10.1242/jeb.245597 PMC10617617

[37] H. M. Belli , C. S. Bresee , M. M. Graff , M. J. Hartmann , PLoS One 2018, 13, e0194981. 29621356 10.1371/journal.pone.0194981 PMC5886528

[38] S. A. Hires , A. Schuyler , J. Sy , V. Huang , I. Wyche , X. Wang , D. Golomb , J. Neurophysiol. 2016, 116, 812. 27250911 10.1152/jn.00511.2015 PMC4995282

[39] D. Voges , K. Carl , G. J. Klauer , R. Uhlig , C. Schilling , C. Behn , H. Witte , IEEE Sens. J. 2011, 12, 332.

[40] R. J. McCormick , in Muscle Foods: Meat Poultry and Seafood Technology, Springer, Boston, MA 1994, 25–62.

[41] K. H. Ha , W. Zhang , H. Jang , S. Kang , L. Wang , P. Tan , H. Hwang , N. Lu , Adv. Mater. 2021, 33, 2103320. 10.1002/adma.202103320 34569100

[42] S. C. Mannsfeld , B. C. Tee , R. M. Stoltenberg , C. V. H. Chen , S. Barman , B. V. Muir , A. N. Sokolov , C. Reese , Z. Bao , Nat. Mater. 2010, 9, 859. 20835231 10.1038/nmat2834

[43] W. Voigt , Lehrbuch der Kristallphysik, Springer Nature, Berlin, Germany 1928.

[44] J. C. Maxwell , A treatise on electricity and magnetism, 1, Clarendon press, Oxford, UK 1873.

[45] J. C. Maxwell , B. Garnett , Phylos. Trans. R. Soc. London. Ser. A 1904, 203, 385.

[46] P. Griffith , J. D. Wallis , “The role of surface conditions in nucleate boiling,” Massachusetts Institute of Technology, Division of Industrial Cooperation, Cambridge, MA, US 1958.

[47] J. S. Woo , J. T. Han , S. Jung , J. I. Jang , H. Y. Kim , H. J. Jeong , S. Y. Jeong , K.‐J. Baeg , G.‐W. Lee , Sci. Rep. 2014, 4, 4804. 24763208 10.1038/srep04804 PMC3999447

[48] J. Cui , H. Mei , J. Zhang , Z. Fan , J. Yang , W. Wang , H. Tohmyoh , X. Mei , MaterialsMaterials 2020, 13, 1290.

[49] D. Zhang , H. Yang , Z. Liu , A. Liu , J. Alloys Compd. 2018, 765, 140.

[50] T. Xu , W. Wang , X. Bian , X. Wang , X. Wang , J. Luo , S. Dong , Sci. Rep. 2015, 5, 12997. 26269285 10.1038/srep12997 PMC4534778

[51] Y. Wan , Y. Wang , C. F. Guo , Mater. Today Phys. 2017, 1, 61.

[52] J. Archard , Proc. R. Soc. Lond. Ser. A 1957, 243, 190.

[53] A. Schallamach , Proc. Phys. Soc. Lond. Sect. B 1952, 65, 657.

[54] V. Gomez , W. Remmas , M. Hernando , A. Ristolainen , C. Rossi , Biomimetics 2024, 9, 83. 38392129 10.3390/biomimetics9020083 PMC10886721

[55] J. Park , M. Kim , J. Park , M. Hong , S. Im , D. Choi , E. Kim , D. Gong , S. Huh , S. U. Jo , C. Kim , Adv. Intell. Syst. 2024, 2300660.

[56] L. Guo , J. Liu , G. Wu , P. Xu , S. Wang , B. Liu , Y. Li , T. Guan , H. Wang , J. Si , T. Du , Sens. Actuators, A 2024, 365, 114875.

[57] H. Varghese , K. V. Priya , U. N. Hareesh , A. Chandran , Macromol. Rapid Commun. 2024, 45, 2300462. 10.1002/marc.202300462 37800886

[58] G. Liu , Y. Jiang , P. Wu , Z. Ma , H. Chen , D. Zhang , Soft Rob. 2023, 10, 97. 10.1089/soro.2021.0166 35483088

[59] S. Harada , W. Honda , T. Arie , S. Akita , K. Takei , ACS Nano 2014, 8, 3921. 24580035 10.1021/nn500845a

[60] X. Hou , L. Xin , Y. Fu , Z. Na , G. Gao , Y. Liu , Q. Xu , P. Zhao , G. Yan , Y. Su , K. Cao , Nano Energy 2023, 118, 109034.

[61] C. Chen , X. L. Li , S. Zhao , Y. Song , Y. Zhu , Q. Wang , C. Zhong , R. Chen , E. Li , Z. Li , J. W. Liu , Device 2023, 1, 100148.

