# Supplementary material for: Whisker‐Implanted Biomimetic Electronic Skin for Tactile Sensing and Blind Perception
Source: Adv Sci (Weinh). 2024 Nov 5;12(2):2408162. doi: 10.1002/advs.202408162 (PMC11727259; doi:10.1002/advs.202408162)
Supplement: Supplementary file 1 — Supporting Information [file ADVS-12-2408162-s002.docx]

**Supporting Information**

**Whisker-Implanted Biomimetic Electronic Skin for Tactile Sensing and Blind Perception**

*Mohammad Zarei^1^, An Woo Jeong^1^, Seung Goo Lee ^*, 1^*

^1^ Department of Chemistry, University of Ulsan, Ulsan 44610, South Korea

**This supporting file includes** Figures S1 to S22, Table S1, Notes S1 to S4,

Videos S1 to S2

■ Author information:

*Corresponding Author: Seung Goo Lee, Email: [lees9@ulsan.ac.kr](mailto:lees9@ulsan.ac.kr)

Seung Goo Lee, ORCID ID: 0000-0002-6504-996X

Mohammad Zarei, ORCID ID: 0000-0002-6351-5058

Department of Chemistry, University of Ulsan, Ulsan 44610, Korea

**Supplementary Figure List**

**Figure. S1** SEM images of the coated leaf skeleton (electrode), show fibrous microstructures and extensive spongy and fibrous networks of the vein (whisker) with an internal channel inside the coated conductive vein.

**Figure. S2** SEM images of the COOH-MWCNT/AgNW-coated leaf-based whisker, demonstrating the whisker diameter variations (where numbers are presented in micrometers).

**Figure. S3** a) Schematic of rat whisker components at base, mid, and tip, demonstrating a Medulla channel inside the vibrissa. b) Schematic of artificial whisker components at the base, mid, and tip, demonstrating the COOH-MWCNT/AgNW-coated surface, porous mid microstructures (mesophyll), and vein channel inside the artificial whisker.

**Figure. S4** Resistance changes of the COOH-MWCNT/AgNW-coated leaf skeleton electrode for different temperatures (measurement interval ~60 sec).

**Figure. S5** Capacitance changes of the COOH-MWCNT/AgNW-coated leaf skeleton electrode for different temperatures (measurement interval ~60 sec).

**Figure. S6** Sheet resistance of electrodes for different COOH-MWCNT loadings in AgNW

solution.

**Figure. S7** Optical transmittance of uncoated leaf skeletons and the COOH-MWCNT/AgNW-coated leaf skeleton electrode.

**Figure. S8** Electrode resistance and capacitance change of the COOH-MWCNT/AgNW-coated leaf skeleton electrode over 30 days.

**Figure. S9** Effect of coating repetition on the sheet resistance of the COOH-MWCNT/AgNW- coated leaf skeleton using a 4-point probe.

**Figure. S10** Capacitive response of the artificial capacitive skins with different center-to-center (CTC) characteristics including samples ES-1, ES-2, and ES-3 with CTC of 140, 160, and 200 µm, respectively.

**Figure. S11** Artificial skin response to the whisker's subtle touch stimulation.

**Figure. S12** Cycling loading of N_2_ gas on the surface of the artificial skin and whisker.

**Figure. S13** Cell design and device configurations for analysis of the artificial skin incorporated robotic rodent to the airstream loading through the escape cell.

**Figure. S14** Stable and reproducible response of artificial skin under cyclic loading and unloading of 8 kPa.

**Figure. S15** Response and relaxation of artificial skin versus time under cyclic loading and unloading for different applied pressures for 4000 cycles (5, 8, and 13 kPa).

**Figure. S16** Stress-strain curve of the artificial skin with different tensile strength regions.

**Figure. S17** Ruggedness of an artificial whisker under severe pressure applied by a motion controller (from left to right, loading and unloading process).

**Figure. S18** Force gauge and motion controller configurations. a) pressure sensing measurement. b) cyclic stability analyses.

**Figure. S19** Physical structure and design characteristics of negative microhoodoo patterns prepared from the positive mold using photolithography.

**Figure. S20** Schematic diagram of the positive microhoodoo PDMS mold fabrication process.

**Figure. S21** Structural design and characteristics of the escape glass cell with dimensions of 30 cm × 15 cm (height × width: 5.2 cm × 5.2 cm).

**Figure. S22** Applying force to the artificial whisker using a force gauge for capacitance and sensitivity measurements.

**Supplementary Table**

**Table. S1** Sensing performance, range, stability, and responsibility of capacitive sensors.

**Supplementary Notes**

**Supplementary Note S1:** Sensitivity measurement of artificial skin.

**Supplementary Note S2:** Thermal conductivity calculation based on the modified Maxwell model.

**Supplementary Note S3:** Influence of hierarchical structures on the sensitivity of the artificial skin.

**Supplementary Note S4:** Test conditions, sensitivity measurement, and minimal detectable force.

**Supplementary Videos**

**Supplementary Video S1:** Artificial skin response to vigorous touch.

**Supplementary Video S2:** Artificial whisker response to low-speed wind.

**Supplementary Figures**


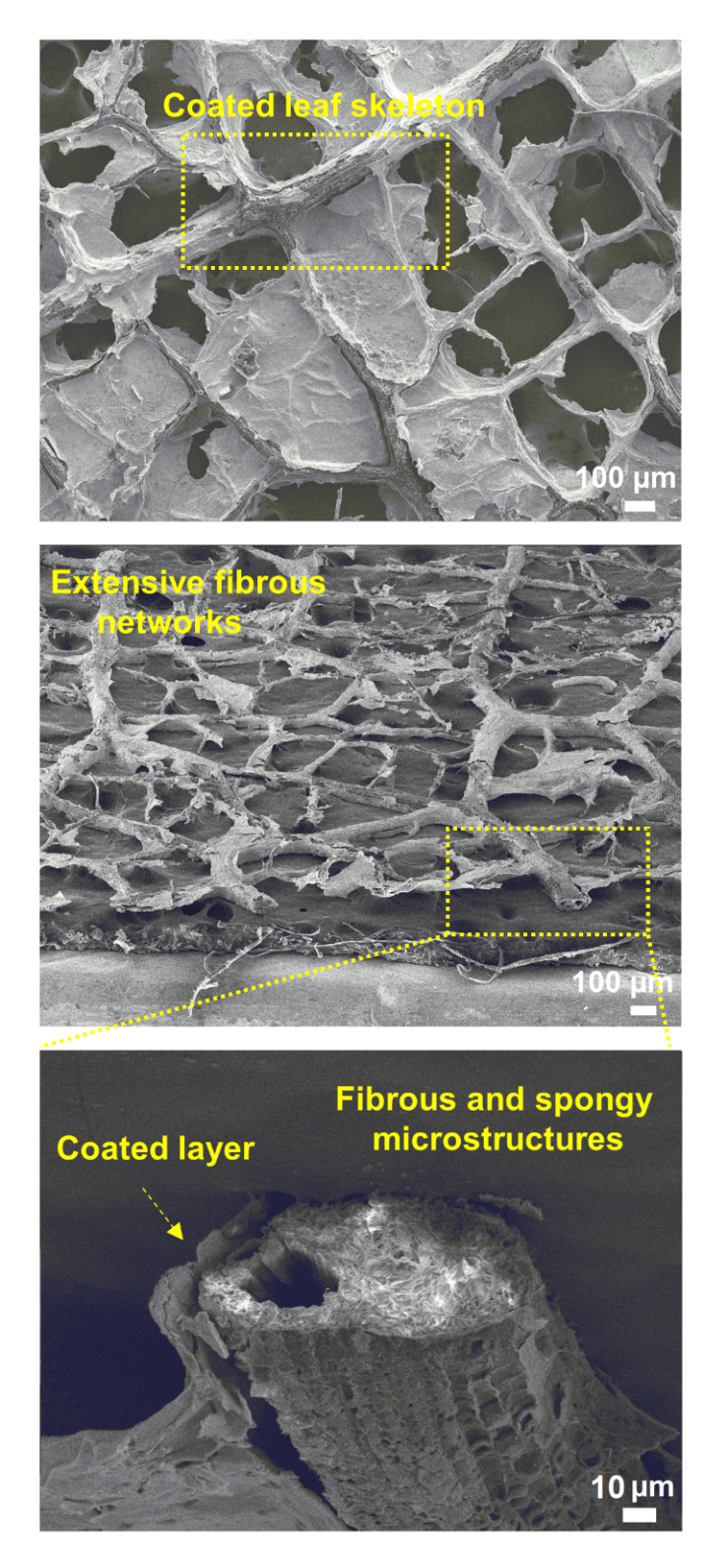


**Figure. S1** SEM images of the coated leaf skeleton (electrode), show fibrous microstructures and extensive spongy and fibrous networks of the vein (whisker) with an internal channel inside the coated conductive vein.


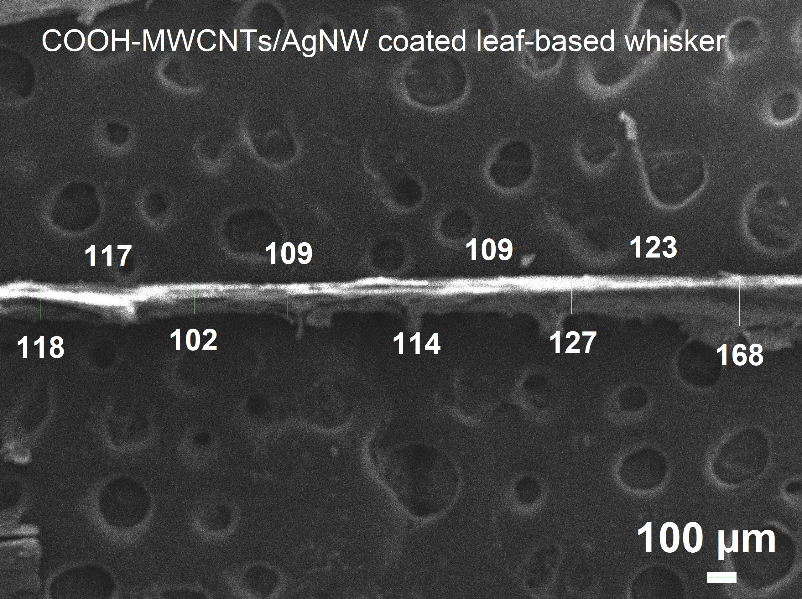


**Figure. S2** SEM images of the COOH-MWCNT/AgNW-coated leaf-based whisker, demonstrating the whisker diameter variations (where numbers are presented in micrometers).


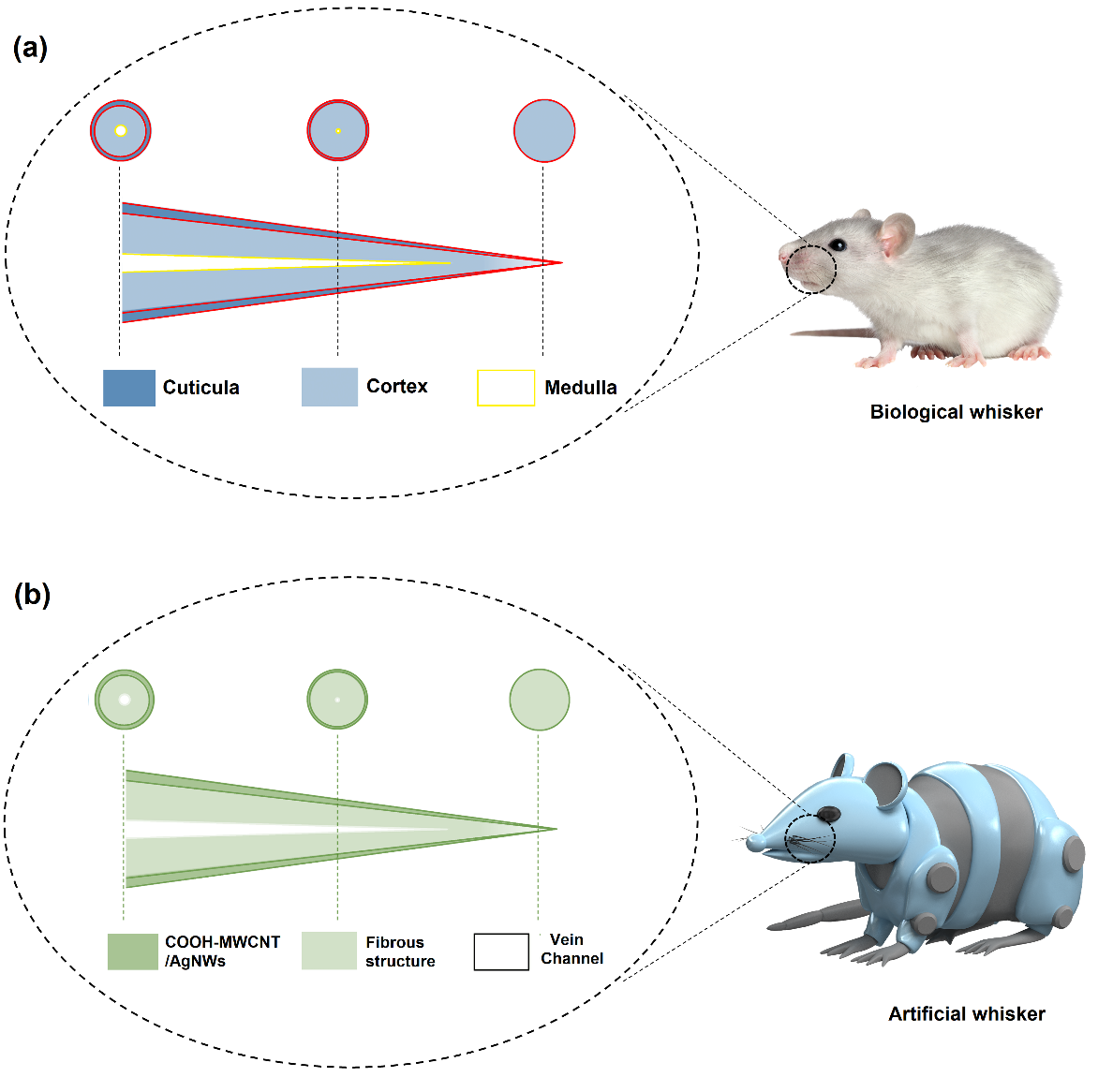


**Figure. S3** a) Schematic of rat whisker components at base, mid, and tip, demonstrating a Medulla channel inside the vibrissa. b) Schematic of artificial whisker components at the base, mid, and tip, demonstrating the COOH-MWCNT/AgNWs coated surface, porous mid microstructures (mesophyll), and vein channel inside the artificial whisker.

**
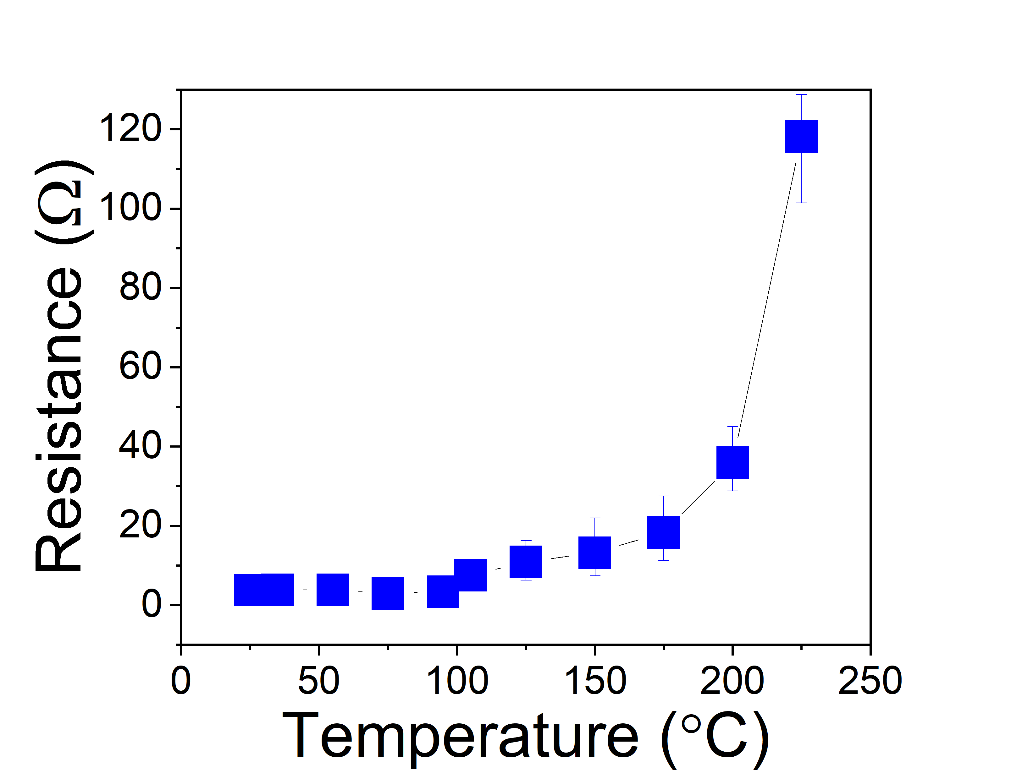
**

**Figure. S4** Resistance changes of the COOH-MWCNT/AgNW-coated leaf skeleton electrode for different temperatures (measurement interval ~60 sec).

**
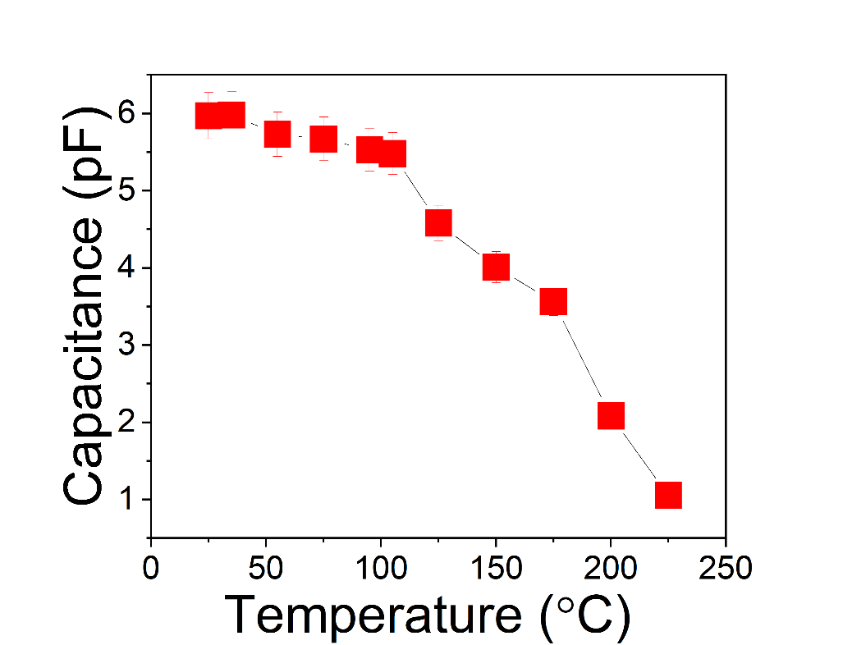
**

**Figure. S5** Capacitance changes of the COOH-MWCNT/AgNW-coated leaf skeleton electrode for different temperatures (measurement interval ~60 sec).


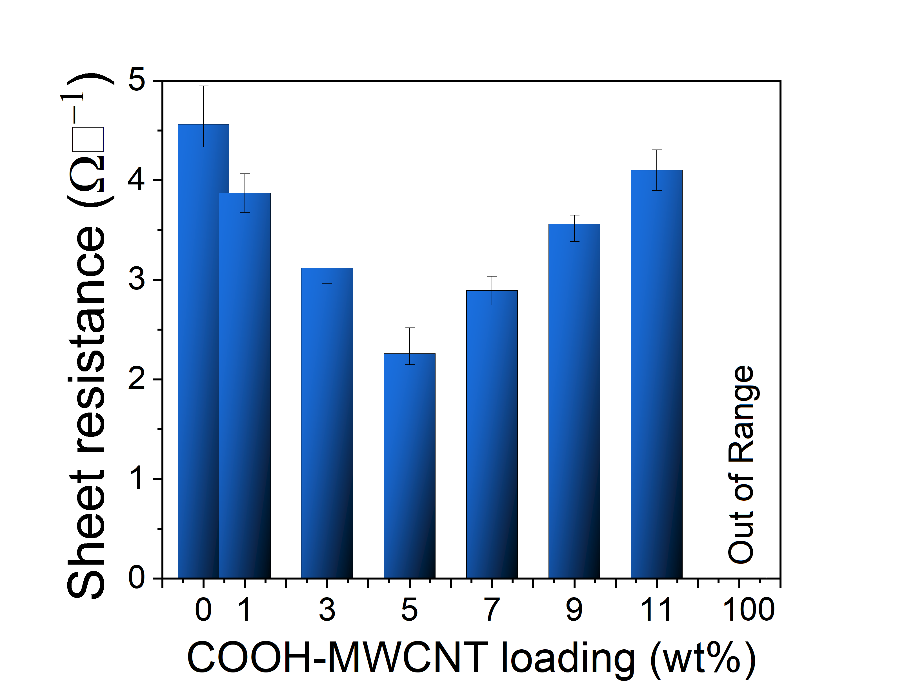


**Figure. S6** Sheet resistance of electrodes for different COOH-MWCNT loadings in AgNW

solution.


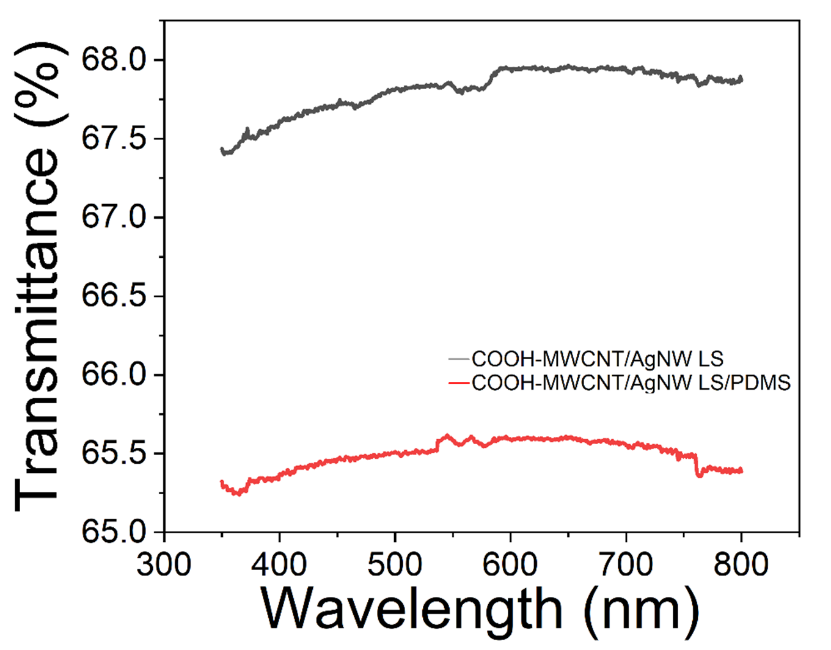


**Figure. S7** Optical transmittance of uncoated leaf skeletons and the COOH-MWCNT/AgNW-coated leaf skeleton electrode.


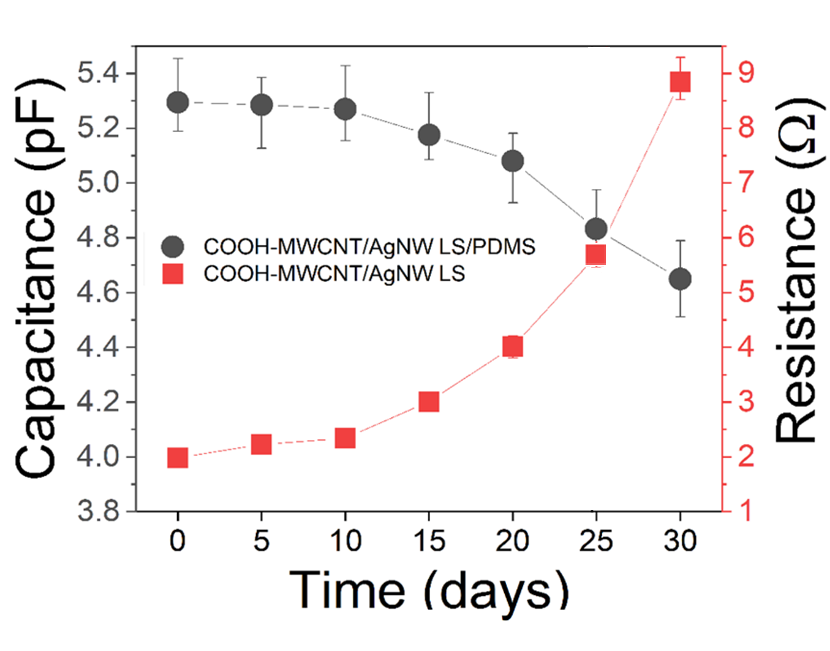


**Figure. S8** Electrode resistance and capacitance change of the COOH-MWCNT/AgNW-coated leaf skeleton electrode over 30 days.


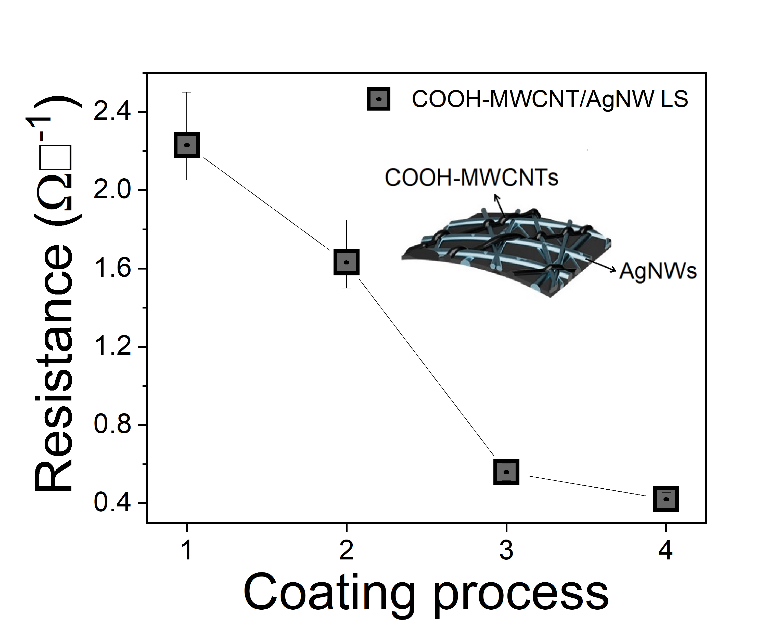


**Figure. S9** Effect of coating repetition on the sheet resistance of the COOH-MWCNT/AgNW- coated leaf skeleton using a 4-point probe.

***
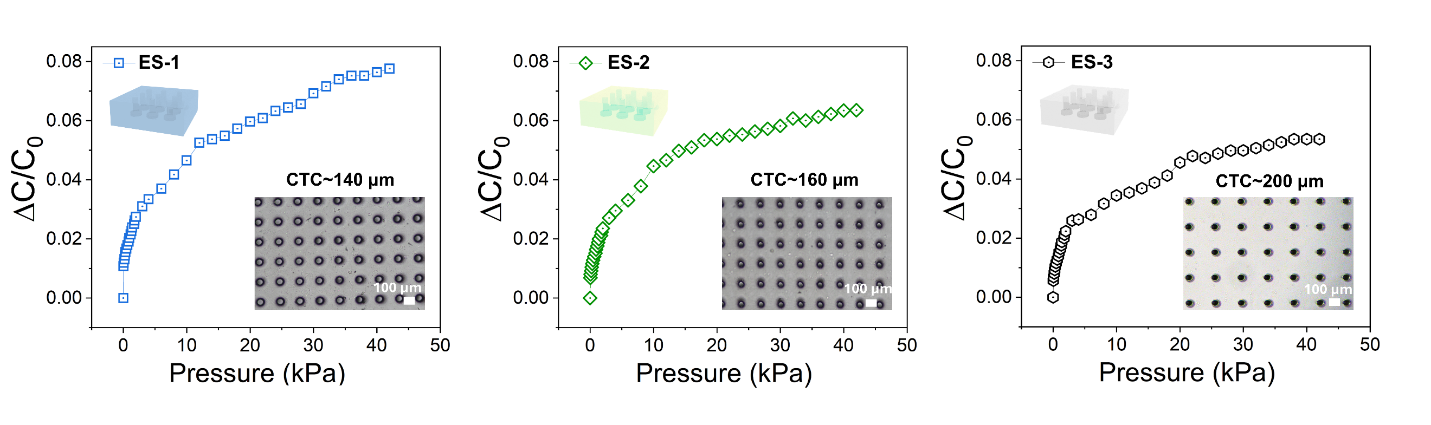
***

**Figure. S10** Capacitive response of the artificial capacitive skins with different center-to-center (CTC) characteristics including samples ES-1, ES-2, and ES-3 with CTC of 140, 160, and 200 µm, respectively.


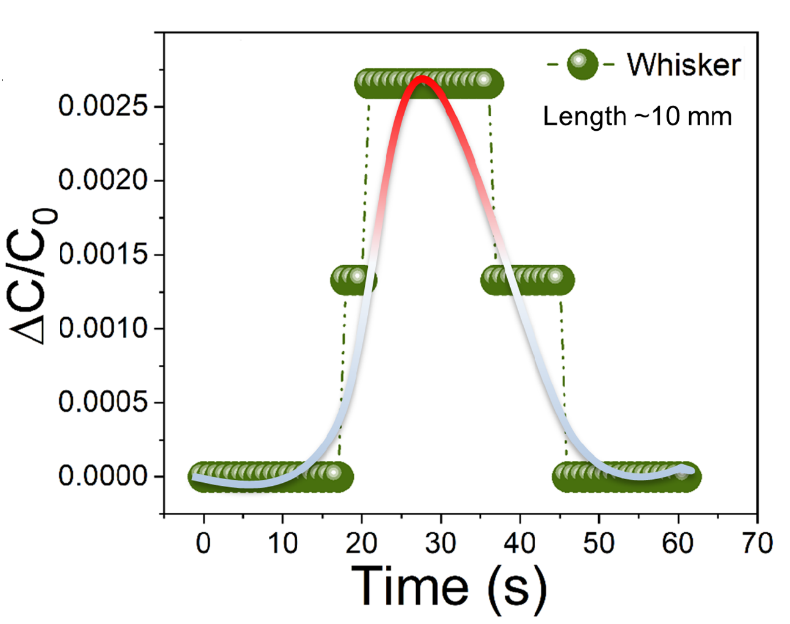


**Figure. S11** Artificial skin response to the whisker's subtle touch stimulation.


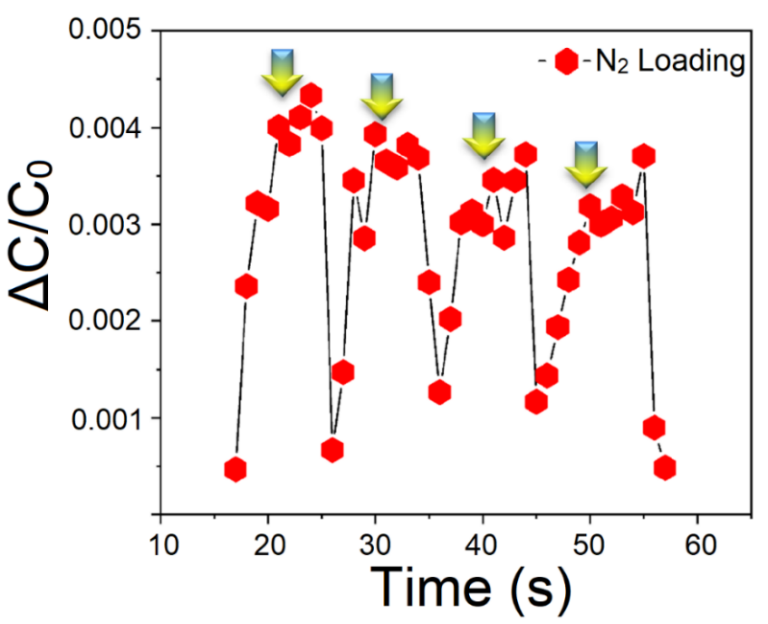


**Figure. S12** Cycling loading of N_2_ gas on the surface of the artificial skin and whisker.


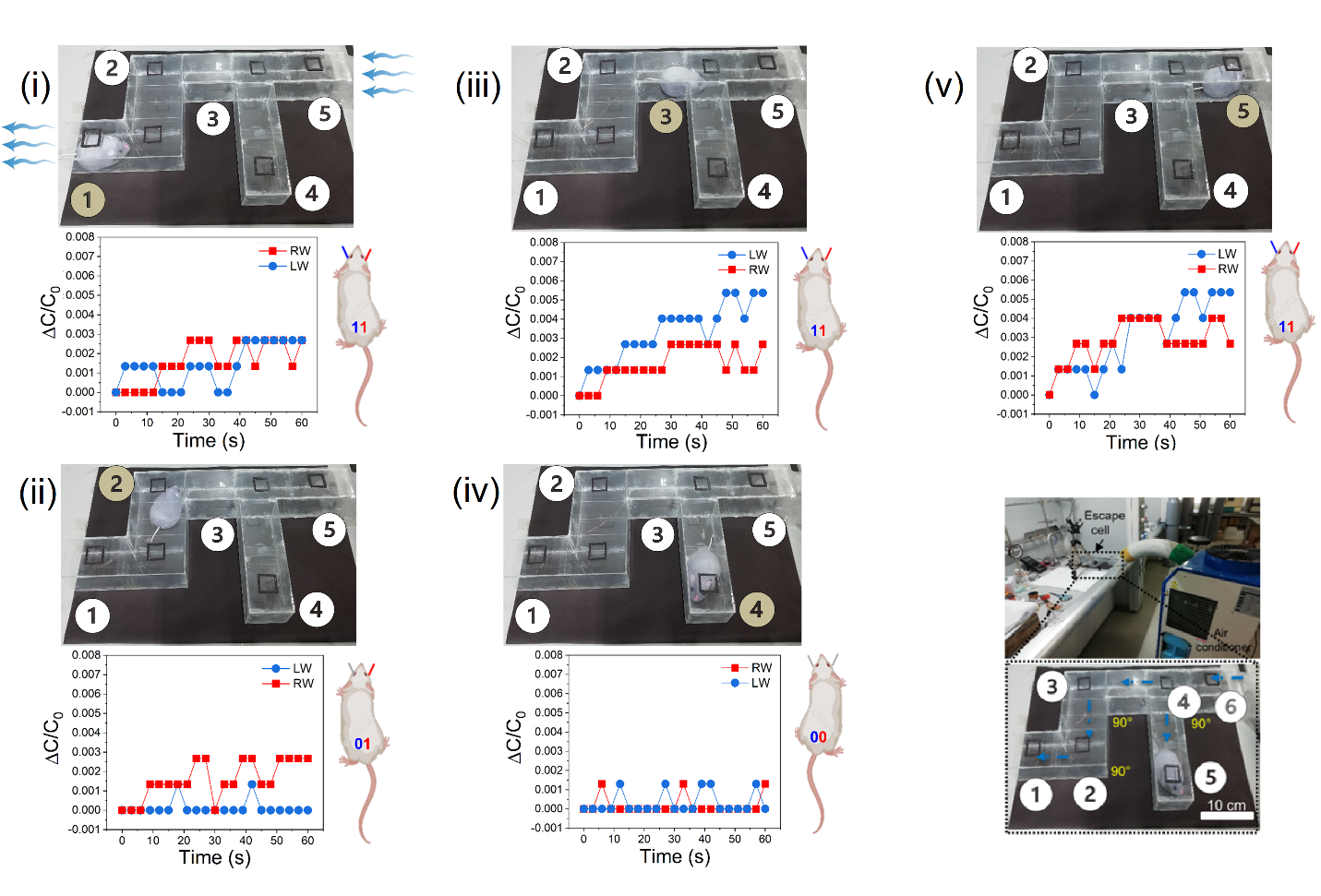


**Figure. S13** Cell design and device configurations for analysis of the artificial skin incorporated robotic rodent to the airstream loading through the escape cell.


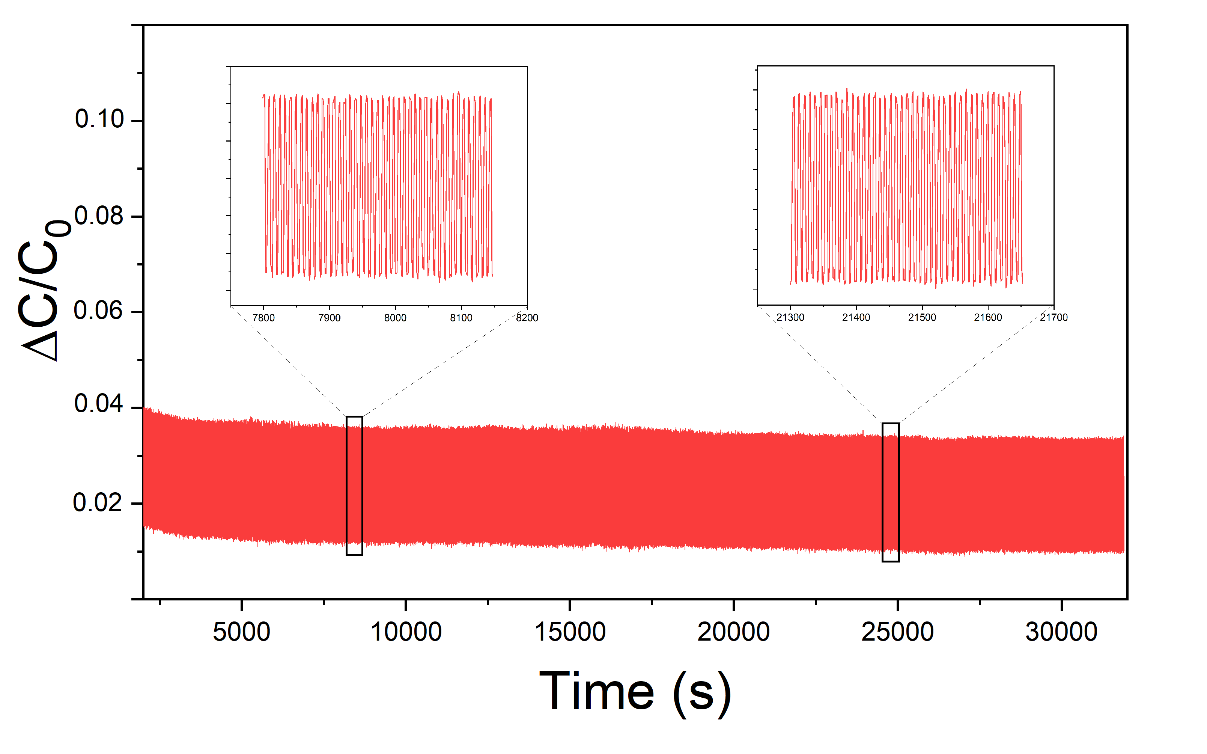


**Figure. S14** Stable and reproducible response of artificial skin under cyclic loading and unloading of 8 kPa.

**
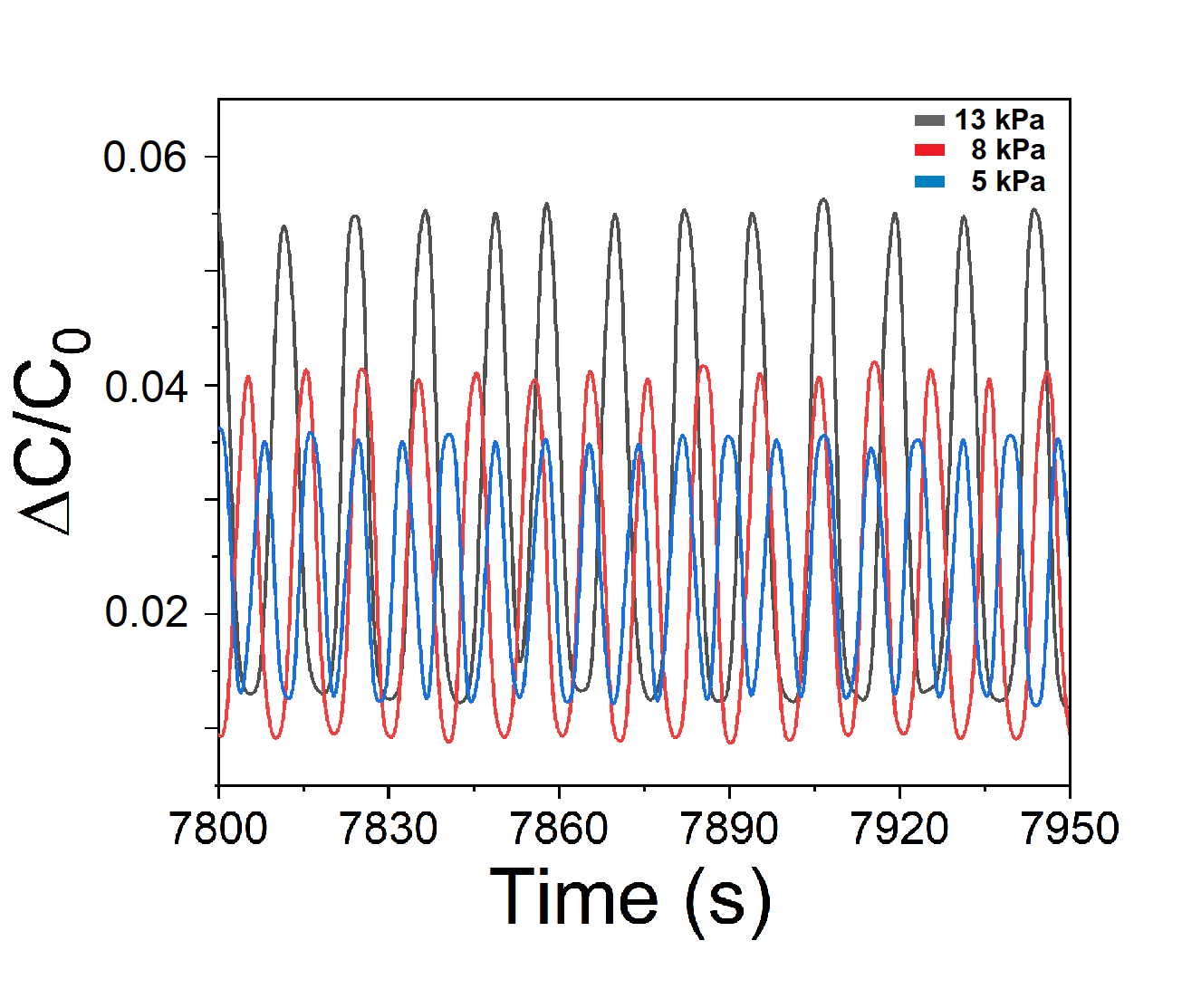
**

**Figure. S15** Response and relaxation of artificial skin versus time under cyclic loading and unloading for different applied pressures for 4000 cycles (5, 8, and 13 kPa).


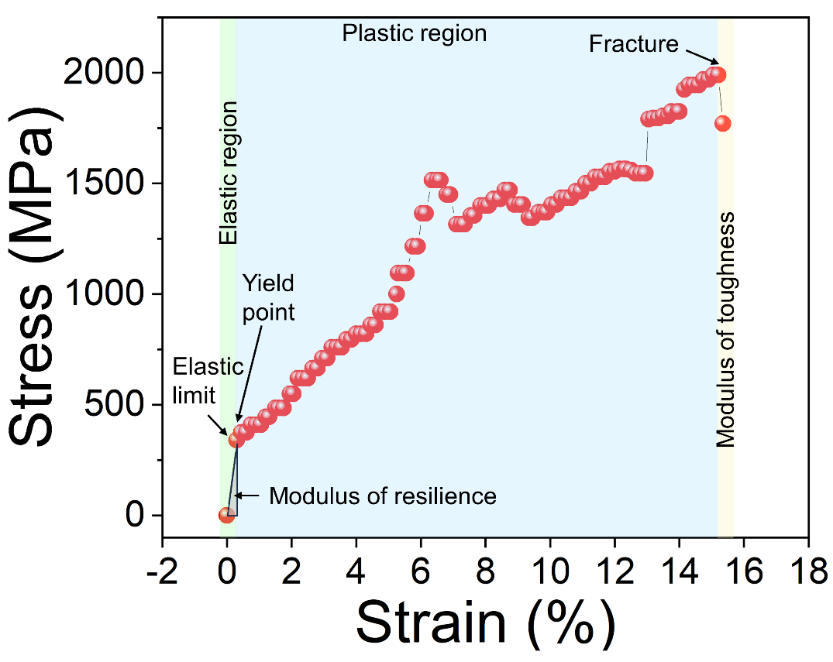


**Figure. S16** Stress-strain curve of the artificial skin with different tensile strength regions.


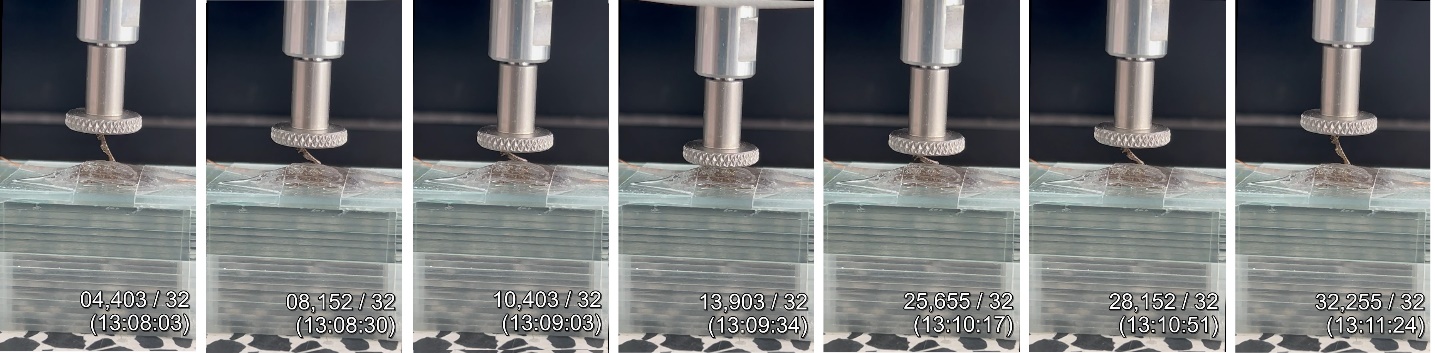


**Figure. S17** Ruggedness of an artificial whisker under severe pressure applied by a motion controller (from left to right, loading and unloading process).


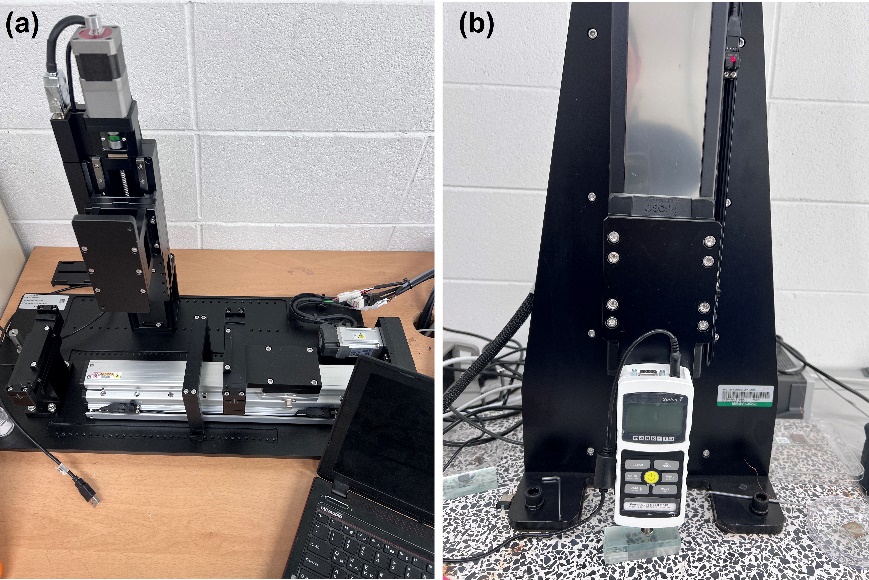


**Figure. S18** Force gauge and motion controller configurations. a) pressure sensing measurement. b) cyclic stability analyses.


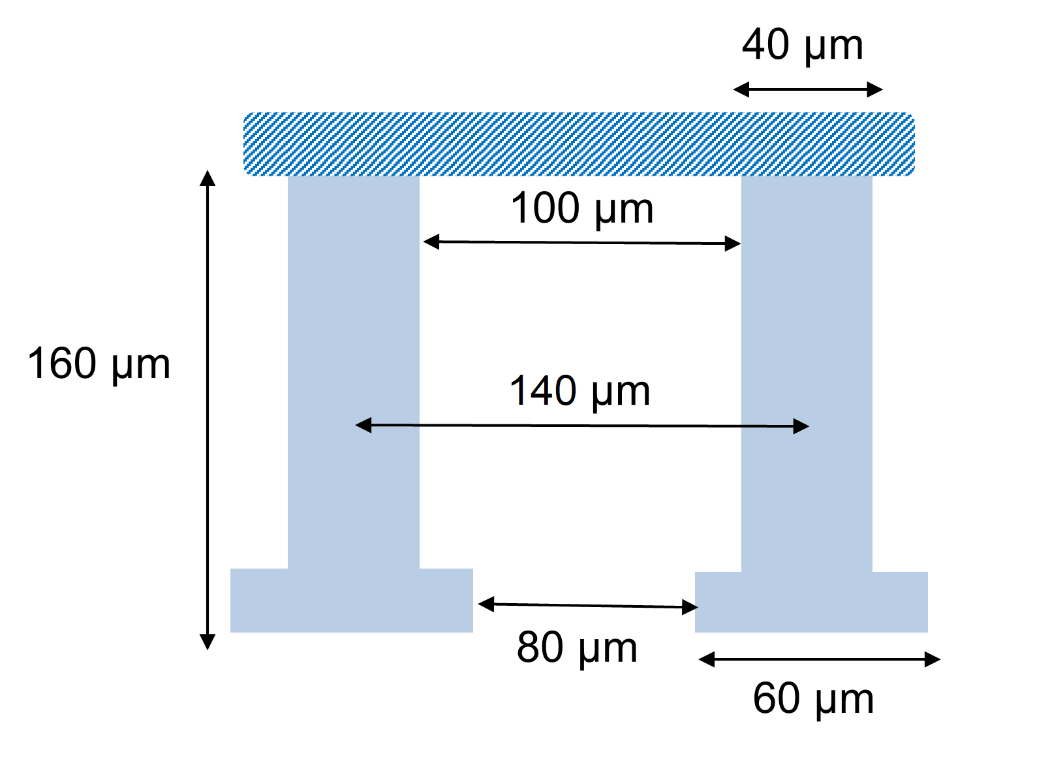


**Figure. S19** Physical structure and design characteristics of negative microhoodoo patterns prepared from the positive mold using photolithography.

**
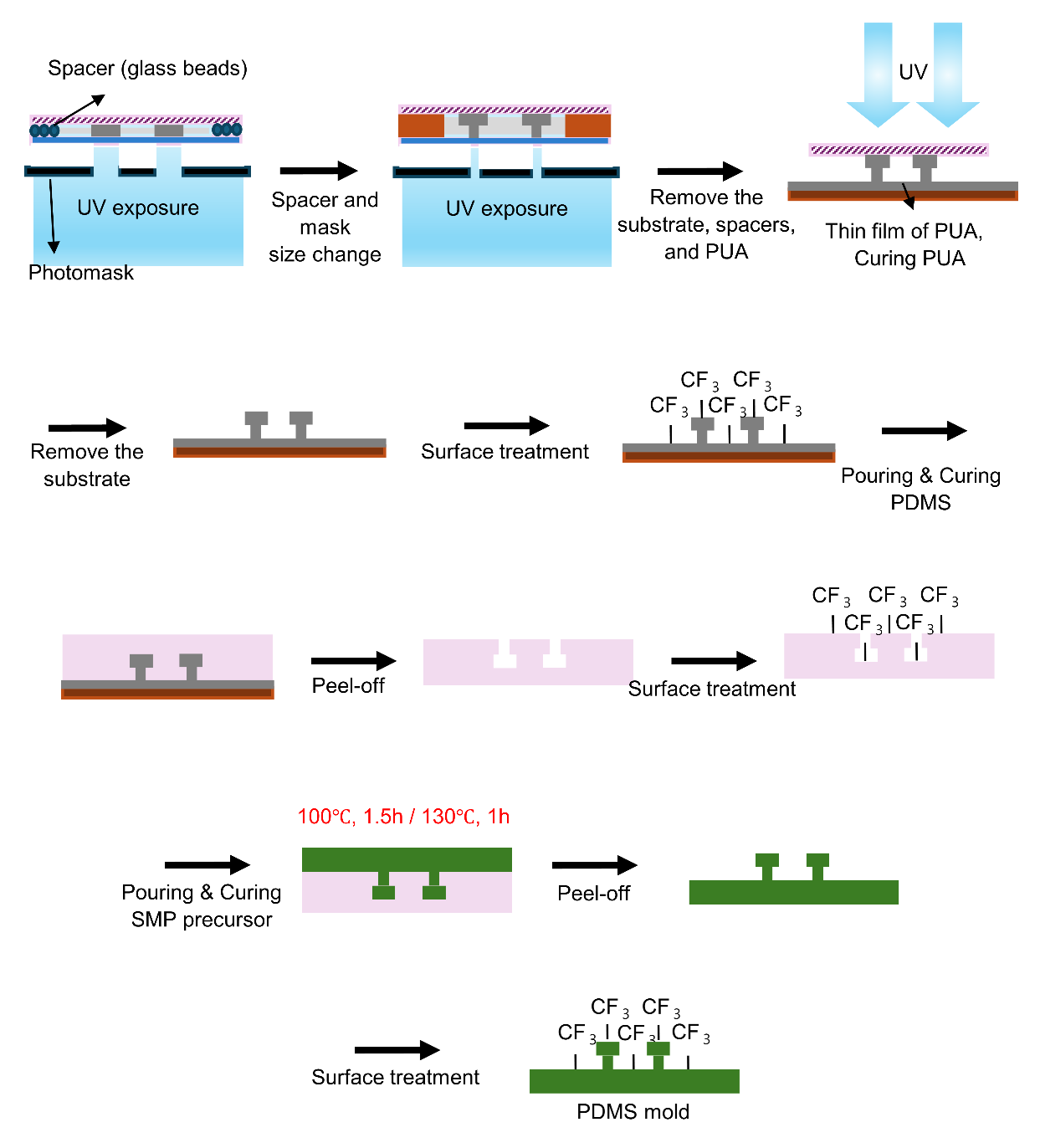
**

**Figure. S20** Schematic diagram of the positive microhoodoo PDMS mold fabrication process.

**
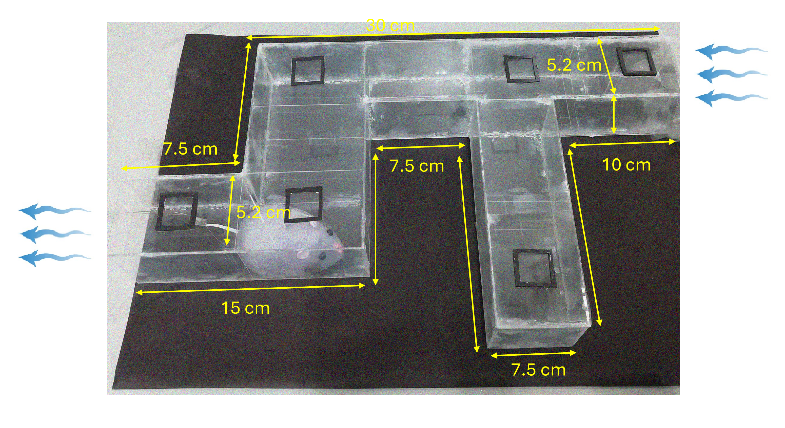
**

**Figure. S21** Structural design and characteristics of the escape glass cell with dimensions of 30 cm × 15 cm (height × width: 5.2 cm × 5.2 cm).

**
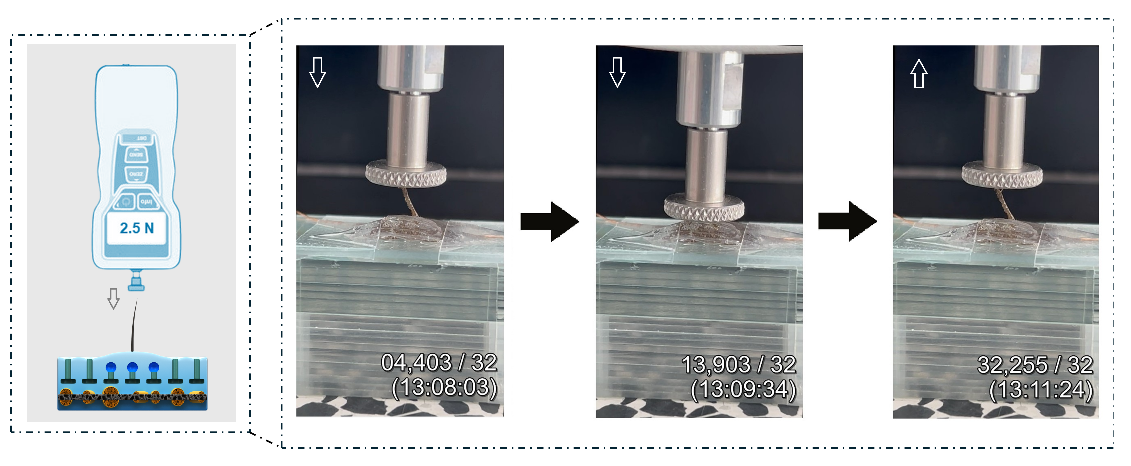
**

**Figure. S22** Applying force to the artificial whisker using a force gauge for capacitance and sensitivity measurements.

**Supplementary Table**

**Table S1** Sensing performance, range, stability, and responsibility of capacitive sensors.

| Materials | Sensitivity &  Performance | Range | Ref | Stability &  Response time |
| --- | --- | --- | --- | --- |
|  |  |  |  |  |
| PVDF, AgNWs@TiO_2_ | 0.0012 kPa^−1^ | ≤1500 kPa | ^[1]^ | 33000 cycles  166.9 ms |
| Graphene oxide, PDMS | 0.627 | 0-3 kPa | ^[2]^ | 240 ms |
| PDMS, Cr/Au | 0.07 kPa^−1^ | 0-4 kPa | ^[3]^ | 1500 cycles  100 ms |
| Graphene, Nylon, PDMS | 0.33 kPa^−1^ | <1 kPa | ^[4]^ | >1000 cycles  <20 ms |
| Silver nanofibers, AgNWs, PEDOT:PSS, Indium gallium zinc oxide | 1.78 × 10^−3^ kPa^−1^  (<350 kPa),  9.65 × 10^−5^ kPa^−1^  (>350 kPa) | 0–1.6 MPa | ^[5]^ | 15000 cycles  32 ms |
| PDMS, PEDOT:PSS, Cu | 0.21 kPa^−1^ | 0–2 kPa | ^[6]^ | 3000 cycles  112 ms |
| CNT, Silicon, Polyhydroxybutyrate-polyhydroxyvalerate (PHB-PHV), Polyurethane | 0.19 ± 0.07 kPa^−1^  (P < 1 kPa),  0.10 ± 0.01 kPa^−1^  (1 < P <10 kPa),  0.04 ± 0.001 kPa^−1^  (10 < P < 20 kPa) | 0–100 kPa | ^[7]^ | 30000 cycles  millisecond range |
| PDMS | 1.2 k Pa^−1^ | <10 kPa | ^[8]^ | 100000 cycles  36 ms |
| POMaC, PGS, PLLA, Magnesium | 0.70±0.40 kPa^−1^  (p<1 kPa)  0.13±0.03 kPa^−1^  (5-10 kPa) | 0-10 kPa | ^[9]^ | 30000 cycles  millisecond |
| PDMS, PEDOT:PSS, cellulose crystals | 2.32 kPa^−1^ | 0-100 kPa | ^[10]^ | 2000 cycles  240 ms |
| PDMS | 0.021 Pa^−1^ | 100-500 Pa | [11] | 100 ms |
| PDMS, Au, Polystyrene | 0.815 kPa^−1^ | 0−50 N | ^[12]^ | 38 ms |
| CNT microarrays | 0.17 kPa^−1^ | 0-25 kPa | ^[13]^ | 10000 cycles  25 ms |
| PDMS, PLA | 0.75 kPa^–1^ | 0-280 kPa | ^[14]^ | 24000 cycles  80 ms |
| CNT, PDMS | 0.034–0.05 kPa ^−1^ |  |  |  |
|  | (P < 0.1 kPa)  0.5 MPa^−1^  (10 kPa < P) | 0-25 kPa | ^[15]^ | 2500 cycles  63 ms |
| Polylactic-co-glycolic acid,  Polycaprolactone | 0.863 ± 0.025 kPa^−1^ | 0-5 kPa | ^[16]^ | 251 ms |
| Rose leaf and petal, AgNW | 0.08 kPa^−1^  (0.007−60 kPa) | 0.1-60 kPa | ^[17]^ | 5000 cycles |
|  |  |  |  |  |
| MXene, thermoplastic polyurethane | 0.24 kPa^−1^ | ≤500 kPa | ^[18]^ | 5000 cycles  62 ms |
| Polyimide (PI), thermoplastic polyurethane nanofiber membrane | 0.28 kPa^–1^  (0–2 kPa) | 0–40 kPa | ^[19]^ | 1000 cycles  65 ms |
| Graphene, TPU | 0.11 kPa^−1^ | 20 Pa-1.4 MPa | ^[20]^ | 4000 cycles  300 ms |
| PDMS, indium-tin-oxide (ITO), polyethylene terephthalate (PET) | 0.017 Pa^−1^  (10-50 Pa)  0.021 Pa^−1^  (100-500 Pa) | 0-54 kPa | ^[21]^ | 120 ms |
| PDMS, RG-067 Silicone | 0.11 kPa^−1^ | ≤300 kPa | ^[22]^ | 409 cycles  21 ms |
| Graphene, PDMS, PI | 0.026 kPa^−1^  (15-40 kPa) | 0-100 kPa | ^[23]^ | 5000 cycles  120 ms |
| PDMS, Polyimide, Au | 3.1 × 10^−3^ kPa^−1^ | 1 MPa | ^[24]^ | 10000 cycles |
| CNT/TPU | 0.04034 kPa^−1^  (0–20 kPa) | 0–800 kPa | ^[25]^ | 60 ms |
| CB/CNTs/PDMS | 3.97 × 10^−3^ kPa^−1^ | 0–600 kPa | ^[26]^ | 1000 cycles  150 ms |
| TPU/ITO/PET | 0.026 kPa^−1^ | 0–13 kPa | ^[27]^ | 5000 cycles  99 ms |
| This study | 0.27 ± 0.09 kPa⁻^1^ | 0–42 kPa |  | 4000 cycles  58 ms |

**Supplementary Notes**

**Supplementary Note S1: Sensitivity measurement of artificial skin**

The relationship between the dielectric constant (*ε*) and capacitance (*C*) is described by the formula for capacitance in a parallel plate capacitor *C*= *ε‧A/d.*^[28]^ The capacitance is directly proportional to the dielectric constant and the area (*A*) of the capacitor, and inversely proportional to the separation between the capacitor plates (*d*). A force gauge-controlled constant speed was used to gradually increase the pressure applied to the e-skin from 0.01 kPa to 46 kPa. The sensitivity is calculated according:

$S=\delta(\Delta C/C_{0})/\delta P$ (S1)

where Δ*C* is the (*C*–*C*_0_) capacitance variation, and P represents applied pressure.^[29]^

**Supplementary Note S2: CNT-based composites' thermal conductivity model**

The modified maxwell expression of the effective thermal conductivity in CNTs-based composites is presented according:^[30]^

$k_{e}=k_{m}\frac{1-f+2f\frac{k_{c}}{k_{c}-k_{m}}\ln\frac{k_{c}+k_{m}}{2k_{m}}}{1-f+2f\frac{k_{m}}{k_{c}-k_{m}}\ln\frac{k_{c}+k_{m}}{2k_{m}}}$ (S2)

where *k_c_* and *k_m_* are the thermal conductivities pertaining to the scattered phase and main phase, respectively, and *k_e_* displays the effective thermal conductivity of two-phase composites. The particle volume fraction of the scattered phase is denoted by *f*.

**Supplementary Note S3: Influence of hierarchical structures on the sensitivity of the artificial skin**

On the basis of contact mechanics models developed by Archard ^[31]^ and Hertz,^[32]^ it is possible to explain the enlarged contact areas of an elastically deformable non-smooth dome with a hierarchical structure. The area of contact with the compressive force increases practically linearly in the Archard model's elastic two-level hierarchical structure, which is made up of tiny protuberances on a hemispherical dome, with a power of 0.89. This response was modeled based on the equation:

*A* = *K W ^0.89^* (S3)

where *A* is the area of contact, *K* is a constant whose value depends on the radii and elastic moduli of the dome and small protuberances, and *W* denotes the compressive force. Similar to the Archard model, the Hertz model is applicable to smooth dome structures without any minor protuberances. According to this concept, the contact area grows with rising applied pressure by a factor of 0.67. According to the Hertz model, the number of contacting tiny protuberances increases along with the deformation of contacting protuberances as the applied pressure is increased in one hierarchical structure. The connection between the total contact area and applied pressure is often roughly linear as the contact area and number of contacting protuberances grow.^[33]^

**Supplementary Note S4: Test conditions, sensitivity measurement, and minimal detectable force**

Different stimuli, such as a finger push versus a whisker touch, can induce varying levels of capacitance change. We also recognize that environmental factors, such as wind and body capacitance, can affect the measured signals. In our study, we aimed to systematically analyze the effects of mechanical stimuli on the artificial skin and whiskers. To clarify our test conditions and provide specific details:

1. Mechanical Stimuli:

*Whisker Sensitivity*: We applied forces ranging from 0 to 21 kPa to the whisker using a controlled force gauge. This setup allowed us to measure the capacitive response of the artificial whisker to different levels of mechanical force.

*Minimum Detectable Force*: We acknowledge that the sensitivity of the whisker to subtle forces needs to be clearly defined. We tested the whisker's response to forces down to the 40 Pa analyzed the signal-to-noise ratio to determine the minimum force that can be distinguished reliably.

1. Wind Stimulation:

*Tunnel Design*: We designed a custom glass escape tunnel with dimensions of 30 cm × 15 cm and a height and width of 5.2 cm × 5.2 cm. The wind speed at the tunnel entrance was controlled and varied between 7.2 and 23.2 m/s. This design aimed to minimize interference and external effects. The minimal detectable wind speed by whisker was 6.7 m/s.

*Airflow Effects*: In natural burrow systems, airflow is typically slow due to small openings and sheltered conditions. We simulated these conditions to understand how low-speed airflow affects the whisker's response. Our setup allows us to control and measure the influence of wind on the whisker accurately and reduce the impact of interference by precise and customized design.

**References:**

[1] R. Han, Y. Liu, Y. Mo, H. Xu, Z. Yang, R. Bao, C. Pan, *Adv. Funct. Mater.* **2023**, *33*, 2305531.

[2] S. Pan, T. Zhang, C. Zhang, N. Liao, M. Zhang, T. Zhao, *Lab Chip* **2024**.

[3] J. Xu, M. Wang, M. Jin, S. Shang, C. Ni, Y. Hu, X. Sun, J. Xu, B. Ji, L. Li, *Nanotechnol. Precis. Eng.* **2024**, *7*.

[4] Z. He, W. Chen, B. Liang, C. Liu, L. Yang, D. Lu, Z. Mo, H. Zhu, Z. Tang, X. Gui, *ACS Appl. Mater. Interfaces* **2018**, *10*, 12816-12823.

[5] B. W. An, S. Heo, S. Ji, F. Bien, J.-U. Park, *Nat. Commun.* **2018**, *9*, 1.

[6] Z. Li, K. Zhao, J. Wang, B. Wang, J. Lu, B. Jia, T. Ji, X. Han, G. Luo, Y. Yu, *ACS Appl. Mater. Interfaces* **2024**.

[7] C. M. Boutry, M. Negre, M. Jorda, O. Vardoulis, A. Chortos, O. Khatib, Z. Bao, *Sci. Rob.* **2018**, *3*.

[8] Y. Wan, Z. Qiu, Y. Hong, Y. Wang, J. Zhang, Q. Liu, Z. Wu, C. F. Guo, *Adv. Electron. Mater.* **2018**, *4*, 1700586.

[9] C. M. Boutry, Y. Kaizawa, B. C. Schroeder, A. Chortos, A. Legrand, Z. Wang, J. Chang, P. Fox, Z. Bao, *Nat. Electron.* **2018**, *1*, 314.

[10] H. Zhang, X. Chen, Y. Liu, C. Yang, W. Liu, M. Qi, D. Zhang, *ACS Appl. Mater. Interfaces*, **2024**, *16*, 2554.

[11] S. Kumar, A. Soni, A. Kumar, *RSC Adv.* **2023**, *13*, 35397.

[12] T. Li, H. Luo, L. Qin, X. Wang, Z. Xiong, H. Ding, Y. Gu, Z. Liu, T. Zhang, *Small* **2016**, *12*, 5042.

[13] Y. Gao, M. Xu, G. Yu, J. Tan, F. Xuan, *Sens. Actuators, A* **2019**, *299*, 111625.

[14] W. Hong, X. Guo, T. Zhang, A. Zhang, Z. Yan, X. Zhang, X. Li, Y. Guan, D. Liao, H. Lu, *ACS Appl. Mater. Interfaces* **2023**, *15*, 46347.

[15] S. Y. Kim, S. Park, H. W. Park, D. H. Park, Y. Jeong, D. H. Kim, *Adv. Mater.* **2015**, *27*, 4178.

[16] M. A. U. Khalid, M. Ali, A. M. Soomro, S. W. Kim, H. B. Kim, B.-G. Lee, K. H. Choi, *Sens. Actuators, A* **2019**, *294*, 140.

[17] A. Elsayes, V. Sharma, K. Yiannacou, A. Koivikko, A. Rasheed, V. Sariola, *Adv. Sustainable Syst.* **2020**, *4*, 2000056.

[18] W. Xu, J. Sun, L. Nie, L. Yang, X. Di, L. Zhang, Q. Zhou, G. Pan, *IEEE Sens. J.* **2024**.

[19] R. Li, M. Panahi-Sarmad, T. Chen, A. Wang, R. Xu, X. Xiao, *ACS Appl. Electron. Mater.* **2022**, *4*, 469.

[20] C. Qu, M. Lu, Z. Zhang, S. Chen, D. Liu, D. Zhang, J. Wang, B. Sheng, *Molecules* **2023**, *28*, 5339.

[21] Bijender, A. Kumar, *Biomed. Mater. Devices* **2023**, *1*, 1009.

[22] T. Hua, Z. Xiang, X. Xia, Z. Li, D. Sun, Y. Wu, Y. Liu, J. Shang, J. Chen, R. Li, *Sensors* **2023**, *23*, 4323.

[23] L. Huang, H. Wang, D. Zhan, F. Fang, *IEEE Sens. J.* **2021**, *21*, 12048.

[24] Y. Tagawa, S. Lee, T. Someya, T. Yokota, *Adv. Electron. Mater.* **2023**, *9*, 2201304.

[25] L. Yang, X. Liu, Y. Xiao, Y. Zhang, G. Zhang, Y. Wang, *Adv. Mater. Technol.* **2023**, *8*, 2201638.

[26] Y. Shi, X. Lü, J. Zhao, W. Wang, X. Meng, P. Wang, F. Li, *Micromachines* **2022**, *13*, 223.

[27] Y. Zhang, M. Gao, C. Gao, G. Zheng, Y. Ji, K. Dai, L. Mi, D. Zhang, C. Liu, C. Shen, *Compos. Sci. Technol.* **2023**, *232*, 109863.

[28] R. B. Mishra, N. El‐Atab, A. M. Hussain, M. M. Hussain, *Adv. Mater. Technol.* **2021**, *6*, 2001023.

[39] Y. Wan, Y. Wang, C. F. Guo, *Mater. Today Phys.* **2017**, *1*, 61.

[30] Q. Xue, *Physica B* **2005**, *368*, 302.

[31] J. Archard, *Proc. R. Soc. London, Ser. A* **1957**, *243*, 190.

[32] A. Schallamach, *Proc. Phys. Soc. London, Sect. B* **1952**, *65*, 657.

[33] G. Y. Bae, S. W. Pak, D. Kim, G. Lee, D. H. Kim, Y. Chung, K. Cho, *Adv. Mater.* **2016**, *28*, 5300.
